# An EPR model for predicting the bearing capacity of single and double-strip foundations near earth slope crests

**DOI:** 10.1371/journal.pone.0301329

**Published:** 2024-05-06

**Authors:** Khalil Sadiq Ismael, Rafi’ Mahmoud Sulaiman

**Affiliations:** Civil Engineering Department, University of Duhok, Duhok, Iraq; Al Mansour University College-Baghdad-Iraq, IRAQ

## Abstract

It is imperative to understand how foundations behave on earthen slopes to accurately predict their allowable carrying capacity in geotechnical engineering. A comprehensive finite element *(FE)* simulation with PLAXIS 2D was conducted to assess the effects of various parameters on the bearing capacity *(BC)* of single- and double-strip foundations placed near the earth’s slope crest. The specified parameters include foundation width *(B)* and depth *(D*_*f*_*/B)*; setback distance between the slope edge and foundation *(b/B)*; soil internal friction *(ϕ)* and cohesion *(c)*; slope inclination (*β*); and spacing between foundations (*S/B*). In addition, the numerically simulated database was used to develop simple mathematical expressions for predicting the capacities in both cases using evolutionary polynomial regression *(EPR)*. The results revealed that the bearing capacity of single- and double-strip foundations increased with an increase in all studied parameters except slope inclination. For single-strip foundations, the outcomes demonstrated that slope inclination has no impact on BC when it is located 6B from the slope edge. However, under interference conditions, the critical center-to-center spacing between foundations is 3–4B, beyond which they behave as individual foundations. Additionally, *EPR* provides a robust method of predicting the *BC* of single- and double-strip foundations within slope crests based on the strong correlation of various statistical criteria between simulated and predicted results from training, validation, and testing. Finally, according to sensitivity analysis, in both single and double-strip foundations resting on an earthen slope crest, *b/B*, *B*, and *ϕ* are the most important input parameters that impact the output results.

## 1. Introduction

In hilly areas, the construction of the foundation on the slope is unavoidable. However, due to fast urbanization and population increase, many structures, such as bridge abutments, electric and mobile transmission towers, buildings, and elevated water tanks, will be near or on the earthen slope crests. As a result, the behavior of the foundation on the slope crest or face will alter in terms of BC and overall structural stability. The passive impedance region towards the slope’s face will decrease based on the foundation’s location from the slope brim, and thereby the carrying capacity of earth slope foundations will be less than that of level ground foundations.

Several methodologies and approaches have been used for estimating the BC of foundations situated on earthen slopes. These strategies include a small-scale laboratory model [1–3], theoretical and analytical research [4–7], and numerical methods [8–10]. However, all of the studies in the literature, in general, concentrated on isolated foundations, and few studies investigated the influence of interfering foundations situated on the slope crest/face by FE analysis [11–13] and analytical analysis [14, 15]. The results showed that with nearby interfering foundations, the failure pattern and associated bearing capacity alter, making typical BC approaches ineffective. Stuart [16] was the first to study shallow foundation interference on level ground and incorporate "efficiency factors" in the BC estimation of two interfering surface strip foundations. Many experimental, analytical, and numerical studies were then done to evaluate the impact of the interfering foundations on level ground [17–21].

As one of the artificial intelligence (AI) techniques, in recent years, EPR has been well adapted to predicting the intricate behavior of most civil engineering problems. This strategy was originally used for environmental modeling by its developers [22–24]. However, because of its superior prediction abilities due to its nature, it has been used in several applications in geotechnical engineering, such as prophesying and assessing foundation settlement [25], compressibility and permeability characteristics of soil [26], sand liquefaction potential [27, 28], and stability analysis [29–31]. Other researchers have used EPR in various geotechnical engineering problems [32–35]. As well as other disciplines of civil engineering, including structural and earthquake engineering [36, 37]. However, according to the literature, only one researcher has used the EPR technique to estimate BC on sloped ground [38], and to date, no research has been conducted using EPR to assess the interference impact on BC caused by strip foundations.

Nowadays, there is no simple and direct approach to estimating the BC of single and double foundations placed on the earth’s slope crest. Therefore, the main goal of this work is to construct simple mathematical expressions for calculating the BC of individual and two interfering strip foundations set on a sloping crest. To achieve this, firstly, a comprehensive numerical analysis utilizing PLAXIS 2D v20 finite element software has been performed. Many geometrical and geotechnical variables were investigated, including footing width (*B*), soil cohesion (*c*), soil friction angle (*ϕ*), footing embedment (*D*_*f*_*/B*), slope angle (*β*), setback distance (*b/B*), and center-to-center footing spacing (*S/B*). Second, the problem was modeled using ERP-MOGA-XL (release 1.0) [39], which operates in a jumbled environment to take advantage of the graphical facilities of Microsoft Excel and the advanced computational MathWorks MATLAB^™^ capabilities to construct mathematical models for BC estimation in a fast and easy manner, greatly assisting design and consulting engineers.

## 2. EPR technique methodology

EPR is a data-driven crossbred approach that integrates numerical regression with genetic programming (GP) effectiveness for constructing uncomplicated and easily explicable mathematical expressions [40]. In general, EPR-MOGA-XL is a two-stage approach; in the first stage, it searches for optimal model structures in polynomial expressions via genetic algorithms (GA) and estimates constant values by using least squares optimization in the second stage [22].

This hybrid strategy has reduced the complications of the estimated expressions as well as the problems related to classic GP. Furthermore, it provides answers in the form of polynomials by limiting the range of operators normally employed in symbolic regressions to the asked subsets. In addition, it outdoes some demerits of other modeling techniques, such as black-box data-driven and physically-based models. One challenge with the former is the unclear mechanisms and difficulty in data collection. The latter, like neural networks, face issues with model identification, over-fitting, and a lack of physical understanding. The EPR can address these issues with an obvious expression for the observed system. Eq (1) presents one of the general structures of the EPR model [22]:

$$Y=a_{o}+\sum_{j=1}^{m}a_{j}.{\left(X_{\text{1}}\right)}^{ES(j,1)}\ldots {\left(X_{k}\right)}^{ES(j,k)}.f\left({\left(X_{1}\right)}^{ES(j,k+1)}\ldots {\left(X_{k}\right)}^{ES(j,2k)}\right)$$ (1)

where ***Y*** is the dependent input variable, ***a***_***o***_ is an optional bias, ***m*** is the target expression terms number, ***a***_***j***_ are constants to be predicted, ***X***_***j***_ are input variables matrices, ***f*** is the type of function defined by the user, ***ES (j*, *z)*** (with ***z*** = 1,…, 2*k*) are the exponents chosen from a user-defined set of candidate values (zero ‘**0**’ should be included).

For the development of the EPR model, Eq (1) is rewritten in vector form as shown in Eq (2) [22]:

$$Y_{N\times 1}\left(\theta ,Z\right)=\left[I_{N\times 1}\;Z_{N\times m}^{j}\right]\times {\left[a_{0}\;a_{1}\;....\;a_{m}\right]}^{T}=Z_{N\times d}\times \theta_{d\times 1}^{T}$$ (2)

where ***Y***_***N×1***_ (***θ***, ***Z***) is the least square estimate vector of *N* target variables, ***θ***_***d×1***_ is the ***d = m+*1** parameters ***a***_***j***_ and ***a***_***o***_ vector, where ***j = 1 to m***; ***Z***_***N×d***_ is a matrix created by unitary (**I**) column vector for bias ***a***_***o***_, and ***m*** is a vector of variables ***Z***_***j***_. This vector for fixed ***j*** is the product of the independent predictor of input variables X = [X_1_ X_2_ X_3_ … X_k_], and k is the variable number.

Generally, the EPR technique uses a dynamic search through a stage-by-stage analogy of regressions with the GA technique. Thus, according to the user-selected function, the search for the best fit starts from Eq 2 and as a result, the matrix of the input variables **X** is as shown in Eq 3.

$$X=\left[\begin{array}{ccccc} x_{11} & x_{12} & x_{13} & .... & x_{1k}\\ x_{21} & x_{22} & x_{23} & .... & x_{2k}\\ x_{31} & x_{32} & x_{33} & .... & x_{3k}\\ .... & .... & .... & .... & ....\\ x_{N1} & x_{N2} & x_{N3} & .... & x_{Nk} \end{array}\right]=\left[X_{1}\;\;\;\;X_{2}\;\;\;\;X_{3}\;\;\;....\;\;X_{k}\right]$$ (3)

where the ***k***^***th***^ column of *X* denotes the candidate variables for the ***j***^***th***^ term in Eq (2). Therefore, the $Z_{N\times m}^{j}$ term in Eq (3) can be written as shown in Eq (4).

$$Z_{N\times 1}^{j}=\left[{\left(X_{1}\right)}^{ES(j,1)}.{\left(X_{2}\right)}^{ES(j,2)}.{\left(X_{3}\right)}^{ES(j,3)}\ldots {\left(X_{k}\right)}^{ES(j,2k)}\right],\forall j=1\ldots m$$ (4)

where ***z***^***j***^ is the ***j***^***th***^ column vector of candidate inputs product, and ***ES***_***k×m***_ is an exponents matrix whose elements are assumed magnitudes within modeler bounds. For example, if candidate exponents for columns in ***X*** are **EX** = [–2, –1, 0, 1, 2], ***m = 5*** (terms number, without bias), and ***k = 5*** is the candidate variables number, then the ***ES*** matrix will be ***5*** × ***5*** in which the columns represent independent variables and the rows represent user-specified terms. So, the exponent ***ES*** matrix will be:

$$ES_{5\times 5}=\left[\begin{array}{ccccc} 1 & 2 & -2 & -1 & 0\\ 0 & 1 & 2 & -2 & -1\\ -1 & 0 & 1 & 2 & -2\\ -2 & -1 & 0 & 1 & 2\\ 2 & -2 & -1 & 0 & 1 \end{array}\right]$$ (5)

Applying Eqs (5) to (4), the following set of expressions is obtained:

$$\begin{array}{cccccccccc} Z_{1}= & {\left(X_{1}\right)}^{1} & {\left(X_{2}\right)}^{2} & {\left(X_{3}\right)}^{-2} & {\left(X_{4}\right)}^{-1} & {\left(X_{5}\right)}^{0}= & X_{1} & X_{2}^{2} & X_{3}^{-2} & X_{4}^{-1}\\ Z_{2}= & {\left(X_{1}\right)}^{0} & {\left(X_{2}\right)}^{1} & {\left(X_{3}\right)}^{2} & {\left(X_{4}\right)}^{-2} & {\left(X_{5}\right)}^{-1}= & X_{2} & X_{3}^{2} & X_{4}^{-2} & X_{5}^{-1}\\ Z_{3}= & {\left(X_{1}\right)}^{-1} & {\left(X_{2}\right)}^{0} & {\left(X_{3}\right)}^{1} & {\left(X_{4}\right)}^{2} & {\left(X_{5}\right)}^{-2}= & X_{3} & X_{1}^{-1} & X_{4}^{2} & X_{5}^{-2}\\ Z_{4}= & {\left(X_{1}\right)}^{-2} & {\left(X_{2}\right)}^{-1} & {\left(X_{3}\right)}^{0} & {\left(X_{4}\right)}^{1} & {\left(X_{5}\right)}^{2}= & X_{4} & X_{1}^{2} & X_{2}^{-1} & X_{5}^{2}\\ Z_{5}= & {\left(X_{1}\right)}^{2} & {\left(X_{2}\right)}^{-2} & {\left(X_{3}\right)}^{-1} & {\left(X_{4}\right)}^{0} & {\left(X_{5}\right)}^{1}= & X_{5} & X_{1}^{2} & X_{2}^{-2} & X_{3}^{-1} \end{array}$$ (6)

Now, substituting the expressions given in Eq (6) into Eq (2) gives

$$\begin{array}{ll} Y & =a_{0}+a_{1}Z_{1}+a_{2}Z_{2}+a_{3}Z_{3}+a_{4}Z_{4}+a_{5}Z_{5}\\ & =a_{0}+a_{1}\frac{X_{1}{X_{2}}^{2}}{{X_{3}}^{2}X_{4}}+a_{2}\frac{X_{2}{X_{3}}^{2}}{{X_{4}}^{2}X_{5}}+a_{3}\frac{X_{3}{X_{4}}^{2}}{X_{1}{X_{5}}^{2}}+a_{4}\frac{X_{4}{X_{5}}^{2}}{{X_{1}}^{2}X_{2}}+a_{5}\frac{X_{5}{X_{1}}^{2}}{{X_{2}}^{2}X_{3}} \end{array}$$ (7)

**Fig 1:**
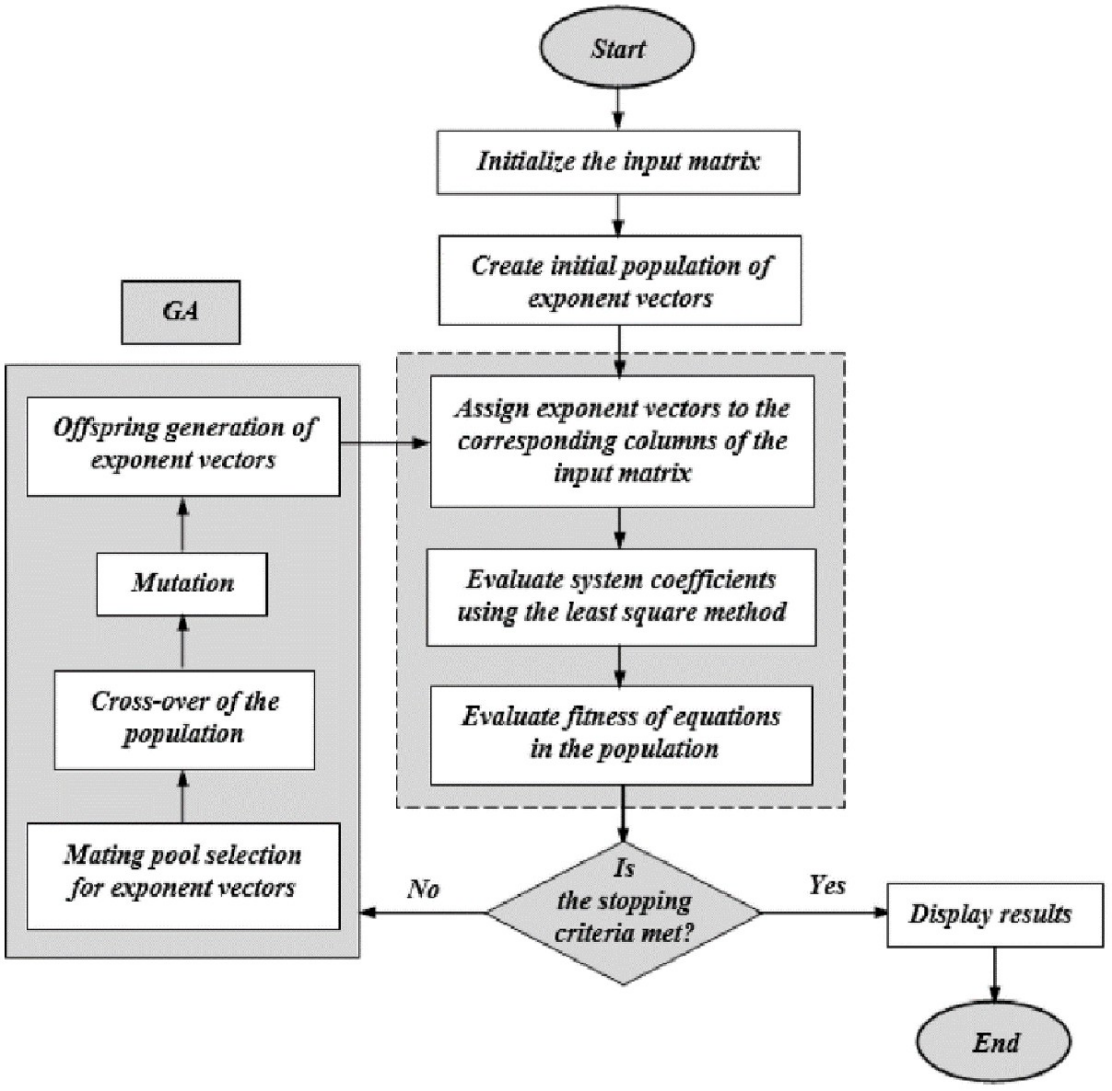
Typical flowchart of EPR-MOGA analysis.

The adjustable parameters *a*_*j*_ then are calculated using linear least squares fitting by reducing the sum of squared errors (**SSE**). Eq (2), determines the ***j***^***th***^ term’s exponents based on each row of **ES** with every exponent in ES corresponding to an EX value. In this way, the EPR, via GA and optimization procedures, searches for the most accurate mathematical model from the **ES** exponents of the studied system. Finally, the best (optimal) model from the proposed EPR model(s) can be chosen based on measuring their fitness and performance during the testing, validation, and training phases using different statistical indicators such as the coefficient of determination (**R**^**2**^), mean absolute error (**MAE**), root mean square error (**RMSE**), variance account for (**VAF**), and **A**^**15**^**-index**. These indices have been used earlier by [41–43] to assess the performance and accuracy of the developed models. The mathematical definitions of these performance indices are presented in Eqs (8)–(12). Fig 1 shows a flow chart of the EPR procedure analysis.


$$R^{2}=1-\frac{\sum_{i=1}^{N}{\left(y_{p}-y_{m}\right)}^{2}}{\sum_{i=1}^{N}{\left(y_{p}-\bar{y}\right)}^{2}}$$ (8)

$$MAE=\frac{1}{N}\sum_{i=1}^{N}\left|y_{p}-y_{m}\right|$$ (9)

$$RMSE=\sqrt{\frac{1}{N}\sum_{i=1}^{N}{\left(y_{p}-y_{m}\right)}^{2}}$$ (10)

$$VAF=\left[1-\frac{\text{var}\;(y_{m}-y_{p})}{var\;(y_{m})}\right]\times 100$$ (11)

$$A^{15}-index=\frac{m15}{N}$$ (12)

In these relations, *y*_*m*_, and *y*_*p*_ represent the simulated and predicted values by the model, respectively, $\bar{y}$ denotes the mean of actual values, m15 shows the sample number that fits the prediction values with a deviation of ±15% compared to measured values, and N is the total number of data points.

## 3. Finite element software and its validation

In this work, PLAXIS 2D v20 software has been used for numerical modeling and analysis. The software is a professional finite element package for deformation and stability analysis issues in geotechnical engineering concerns such as tunnels, earth structures, deep excavations, etc. Many researchers have successfully utilized this program to explore foundation behavior on earth slopes, bearing capacity, and slope stability evaluation [44–46]. The findings of experimental works described by [17, 47, 48] and numerical analysis using PLAXIS 3D performed by [11, 49] were used to verify the FE model. Then, the validated model was used for the parametric analysis for predicting the bearing pressure of single and two-closely spaced strip footings positioned at varied locations from the crest of an earth slope.

### 3.1 Model boundaries

**Fig 2:**
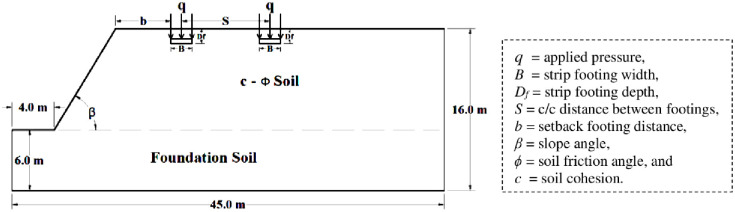
Model geometry.

In this work, the size of the slope model geometry has been set to ensure that isobar stresses do not reach the model’s borders, as illustrated in Fig 2. The model boundaries have been selected with the bottom boundary as fully rigid and restricted in both directions, vertically and horizontally, whereas the vertical boundaries are fixed only horizontally, but vertical deformation is allowed, while the slope face is kept free of movement.

### 3.2 Mesh sensitivity

**Fig 3:**
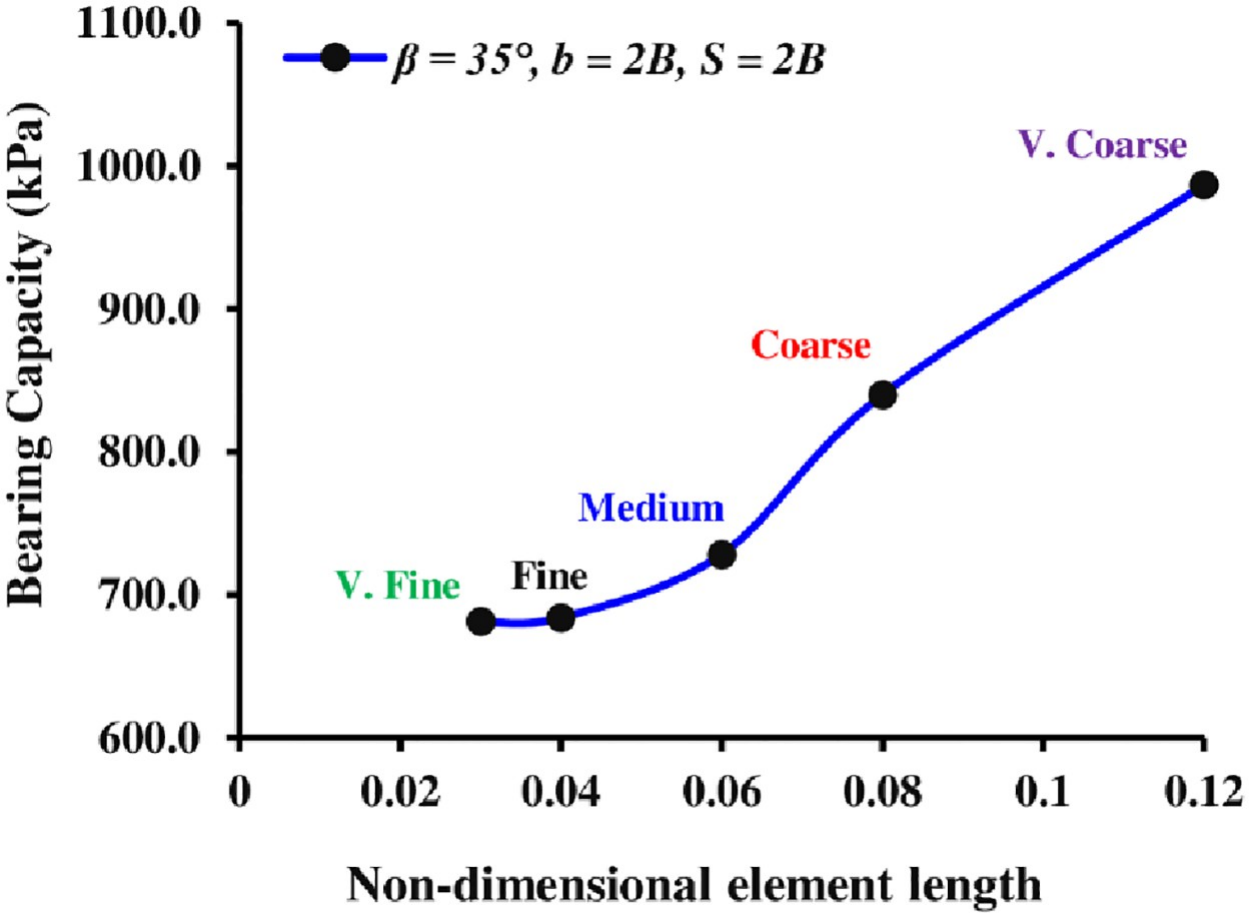
Mesh sensitivity.

**Fig 4:**
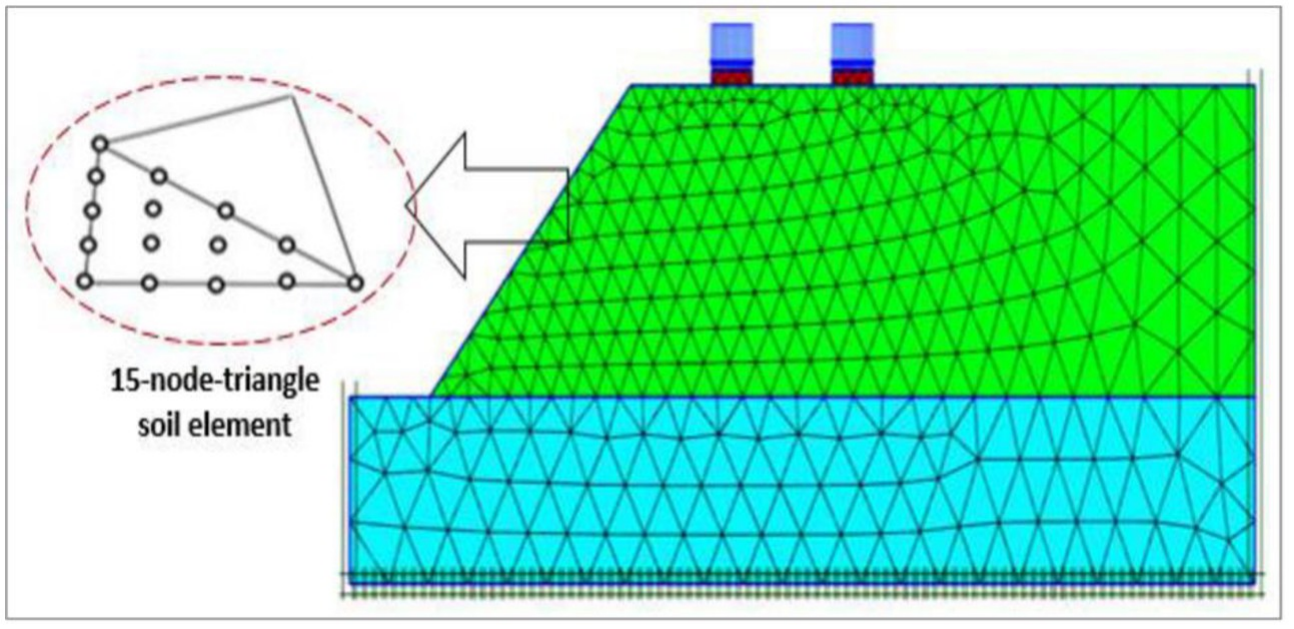
Mesh generation and boundary fixation.

The entire model has been divided into finite elements to conduct numerical analyses with a reliable mesh. A coarse mesh makes it difficult to capture crucial soil and foundation characteristics, and a mesh with a very fine size produces a large number of elements and needs more processing time. Therefore, a sensitivity analysis was conducted to find the ideal mesh element size for the FE model, considering the available meshing types in PLAXIS,”i.e., very fine, fine, medium, coarse, and very coarse based on their mesh coarseness factor”. To diminish the mesh’s reliance on the numerical model, the best mesh element size is determined in terms of its non-dimensional average element size (NAES). Fig 3 exhibits the impact of the elements’ size on the bearing pressure of the continuous foundation lying on the earth’s slope. As a result, a fine meshing approach (NAES = 0.04) indicated suitable outcomes and thus has been used for the current numerical model study. After properly defining the soil and foundation, loads, and boundaries of the slope geometry model, a fully automatic mesh generation is conducted. Fig 4 depicts the geometry and meshing of the 2D FE model.

### 3.3 Constitutive modeling

**Fig 5:**
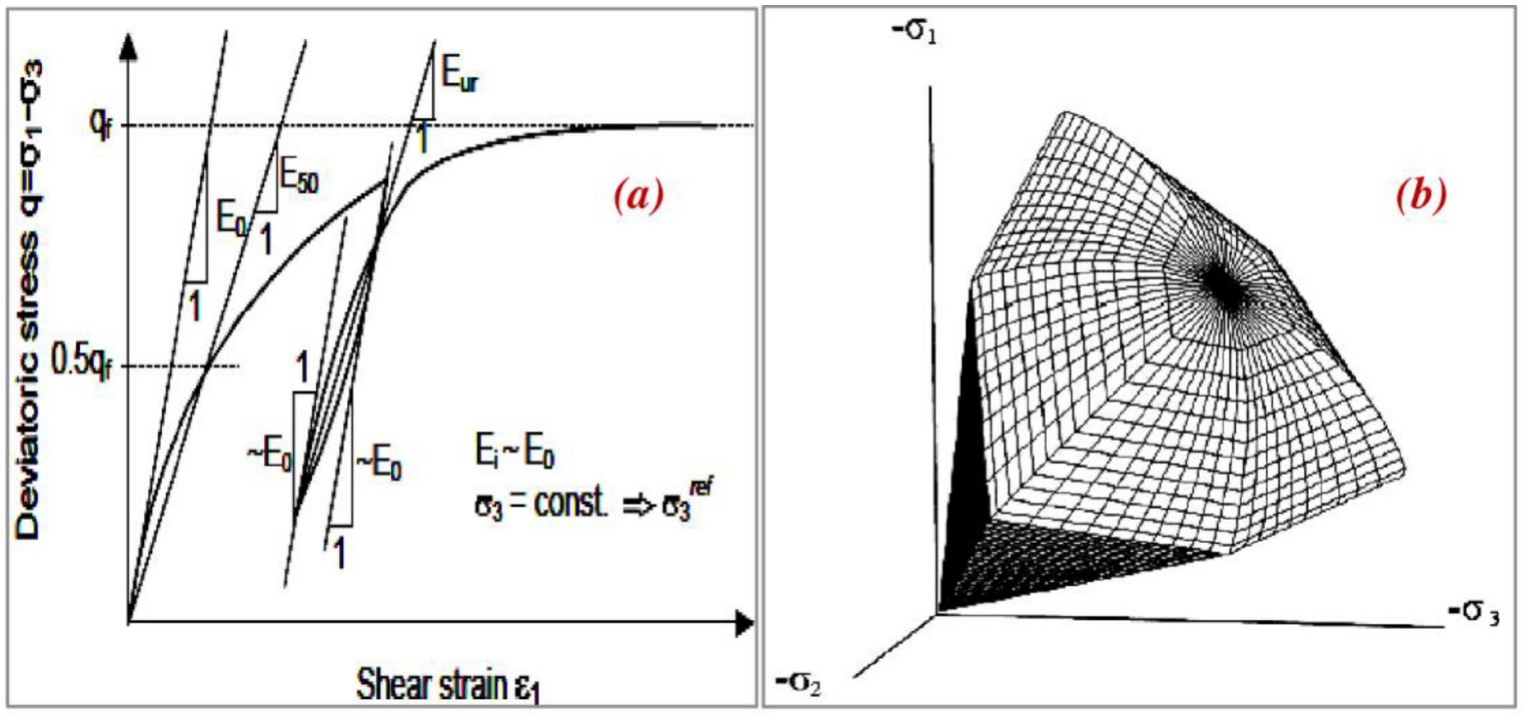
(a) Different moduli of typical stress-strain HS small soil model, (b) The cap yield surface in principle stress space.

Both the backfill and foundation soils are simulated using the hardening soil model with small-strain stiffness (HSsmall). This model is an extension of the hardening soil (HS) model that takes into account increasing soil stiffness at low strains. This behavior is given in the HSsmall model by two extra material parameters, ${\text{G}}_{0}^{\text{ref}}$, and γ_0.7_ where ${\text{G}}_{0}^{\text{ref}}$ is the small-strain shear modulus and γ_0.7_ is the strain level at which the shear modulus has decreased to approximately 70% of the small-strain shear modulus. This model has a cap yield surface (see Fig 5) and can more accurately reproduce soil deformations than the HS, linear elastic (LE), and elastoplastic Mohr-Coulomb (MC) because of its non-linear stress-strain relationship and soil stiffness calculated using an oedometer loading tangent stiffness, triaxle unloading/reloading stiffness, and triaxle loading secant stiffness [50, 51].

In this study, the soil elements are modeled using 15-node triangular elements with two translational degrees of freedom per node that contain 12 stress points. This is because it converges more quickly and gives more accurate results of the stresses and deformations than the 6-noded elements. While the LE model has been used for modeling the concrete foundation, Table 1 shows the soil and concrete footing parameters used in the current numerical study.

**Table 1:** Soil and concrete footing properties.

Parameters	Name	Backfill c- *ϕ* Soil	Foundation Soil	Concrete Foundation
Material model	-----	HS_small_	HS_small_	Linear elastic
Material behavior type	-----	Drained	Drained	Non-porous
Unsaturated unit weight, kN/m^3^	*γ*_*unsat*_	16	20	24
Saturated unit weight, kN/m^3^	*γ*_*sat*_	18	20	
Secant stiffness in standard drained triaxial test, kN/m^2^	*E*_*50*_^*ref*^	3.0×10^4^	3.0×10^4^	
Tangent stiffness for primary oedometer loading, kN/m^2^	*E*_*oed*_^*ref*^	3.601×10^4^	3.0×10^4^	25×10^6^
Unloading/reloading stiffness, kN/m^2^	*E*_*ur*_^*ref*^	1.108×10^5^	1.2×10^5^	
Power for a stress-level dependency on stiffness	*m*	0.5	1	
Cohesion, kN/m^2^	*c*_*ref*_	8, 15, and 20	40	
Friction angle ^(o)^	*ϕ*	32, 36 and 40	24	
Dilatancy angle ^(o)^	*ψ*	(*ϕ*– 30)	0	
Shear strain at which *Gs =* 0.722 *G*_*0*_	*γ*_*0*.*7*_	1.5×10^−4^	1.0×10^−3^	
Shear modulus at very small strains, kN/m^2^	*G*_*0*_^*ref*^	1.0×10^5^	1.0×10^5^	
Poisson’s ratio	*ν′*_*ur*_	0.2	0.2	0.15

### 3.4 Finite element model validation

It is assumed that the experimental results represent the real behavior of the object under test, with specific measuring errors due to devices, human force, bad material,… etc. However, these errors should be bound and lies within certain margin. On the other side the simulation results represent the behavior of the same object based on its theoretical model. Generally, the experimental results are more precise/accurate compared to any results obtained from any model utilizing any software. Obtaining the correct results from a experimental set up is a challenge to any researcher.

**Fig 6:**
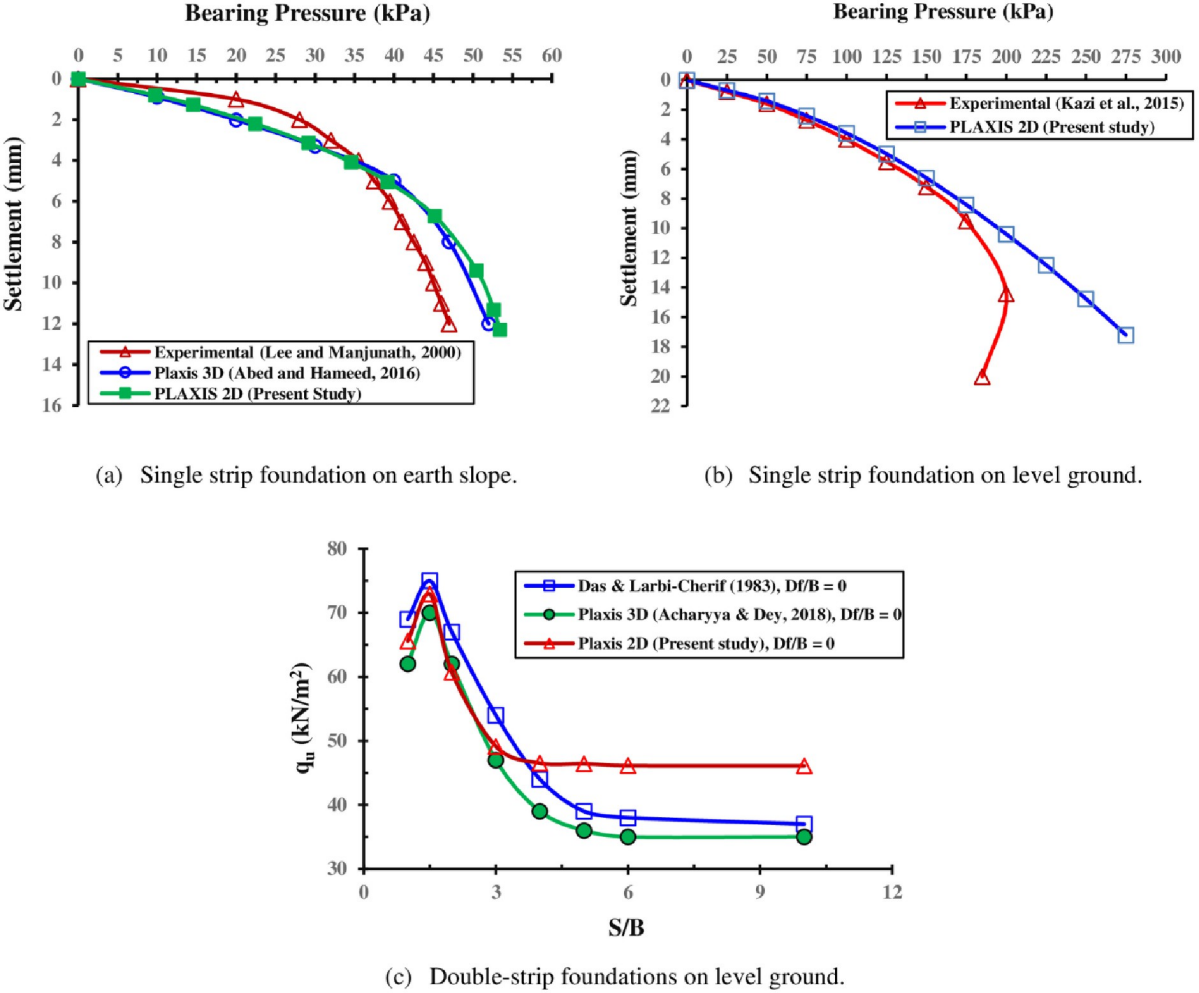
Verification of the numerical model.

To ensure PLAXIS accuracy in analysis, the numerical model findings were compared to those of experimental work completed by Lee and Manjunath [47] and numerical work utilizing PLAXIS 3D code by Abed and Hameed [49] for a strip foundation set on an earthen slope. For further examination of the FE model with more cases and data, the 2D numerical outcomes were also compared with two different experimental works conducted by Kazi et al. [48] and Das and Larbi-Cherif [17] for single and double strip footings on level ground, respectively. In these analyses, the FE mesh is set to "fine" with a non-dimensional average element size NAES ≈ 0.025–0.050. The results were compared through load-settlement curves or the ultimate capacity of footings against different center-to-center spacing in Fig 6. As seen the current model outcomes closely match experimental results in terms of both magnitude and trend with acceptable difference. The discrepancy between simulation and experimental results can be attributed to the difference between the real object and its assumed model either physical or mathematical description specially if the other error sources are minimized and others, due to the numerical model realized, such as: validation of experimental measurements; the boundary conditions for the experimental tests corresponding to the model realized; and the correct choice of coefficients in the numerical constitutive model used with what is suitable for experimental tests. Consequently, the current model can be used for conducting a parametric study to explore the numerous design parameters’ impact on the bearing pressure of a single and two closely interfering strip foundations situated on a sloped soil surface.

## 4. Results and discussion

### 4.1 Numerical results of single-strip foundation

Table 2 summarizes the geometrical and geotechnical soil variables used in this study to compute the normalized bearing capacity (*q*_*u*_/*γ H*) of a strip foundation positioned on an earthen slope crest model outlined in Fig 3. The statistical characteristics of input and target data are presented in Table 3.

**Table 2:** Used parameters in the numerical analysis of single strip foundation.

		*q*_*u*_ */γH*			
*B (m)*	*c (kPa)*	*ϕ°*	*D*_*f*_ */B*	*β°*	*b/B*
					0.0
					1.0
	8	32	0.0	35	2.0
1.0	15	36	0.5	40	3.0
2.0	20	40	1.0	45	4.0
				50	5.0
					6.0
					7.0

**Table 3:** Statistical information of input and target data used in the EPR model for single strip foundation.

	Minimum	Maximum	Mean	Median	Std. deviation
*B (m)*	1	2	1.5	1.5	0.500
*c (kPa)*	8	20	14.333	15	4.923
*ϕ°*	32	40	36	36	3.267
*D*_*f*_ */B*	0	1	0.5	0.5	0.408
*β°*	35	50	42.5	42.5	5.592
*b/B*	0	7	3.5	3.5	2.292
*q*_*u*_ */γH*	0.285	5.548	2.719	2.635	1.022

#### 4.1.1 Effect of foundation width

**Fig 7:**
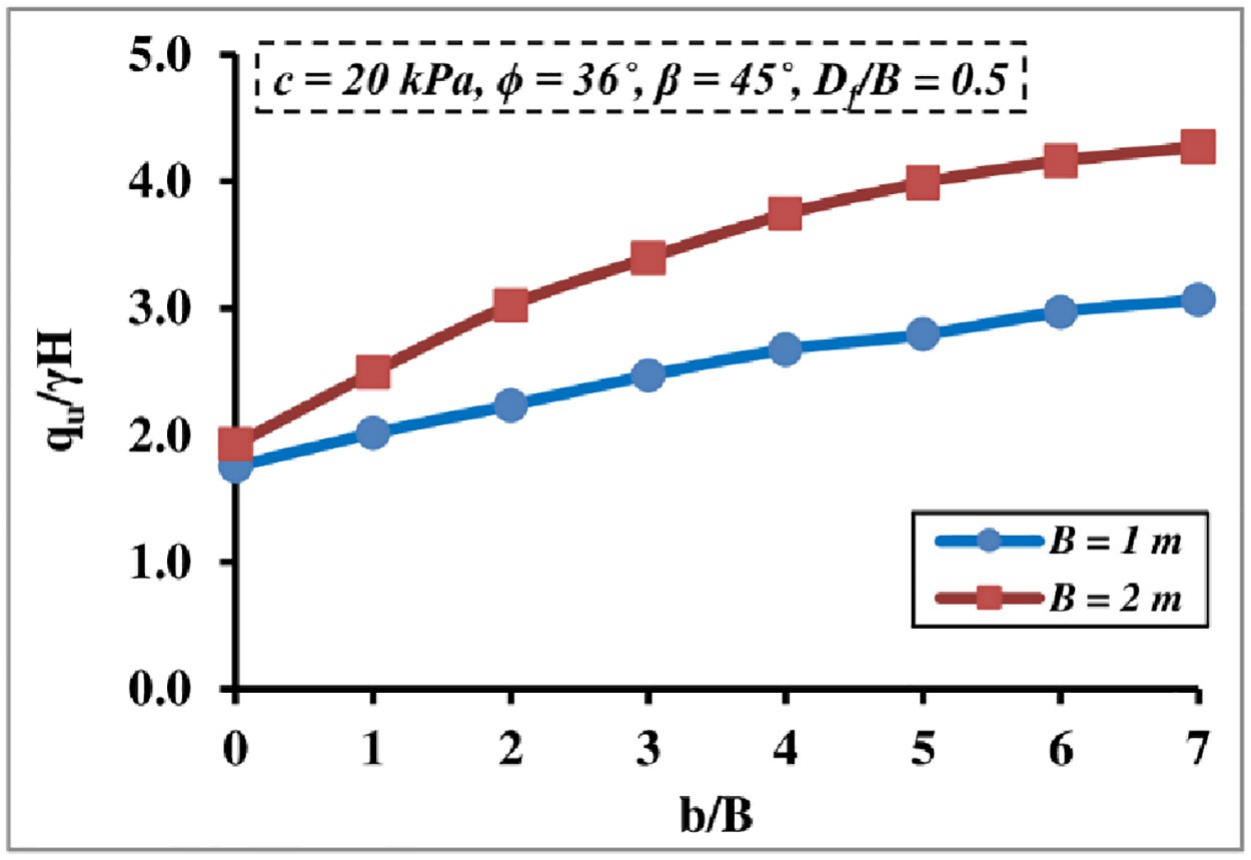
Effect of *B* and *b/B* ratio on *q*_*u*_*/γH*.

Fig 7 displays the combined impact of the foundation width *B* and *b/B* ratio on the *q*_*u*_/*γ H* value; it reveals that both have a considerable influence on the *q*_*u*_/*γ H* magnitude. The results observed that when the *B* and *b/B* ratio increased, so did the *q*_*u*_/*γ H* value, since a higher soil depth beneath the foundation contributed to its ability to sustain the applied load. Because soil collapse at a small *b/B* ratio is caused by a combination of bearing capacity and slope instability failure, the influence is more noticeable for a larger *b/B* ratio; however, this increase in *q*_*u*_/*γ H* value diminishes after *b/B* = 6.

#### 4.1.2 Effect of slope angle and foundation setback

**Fig 8:**
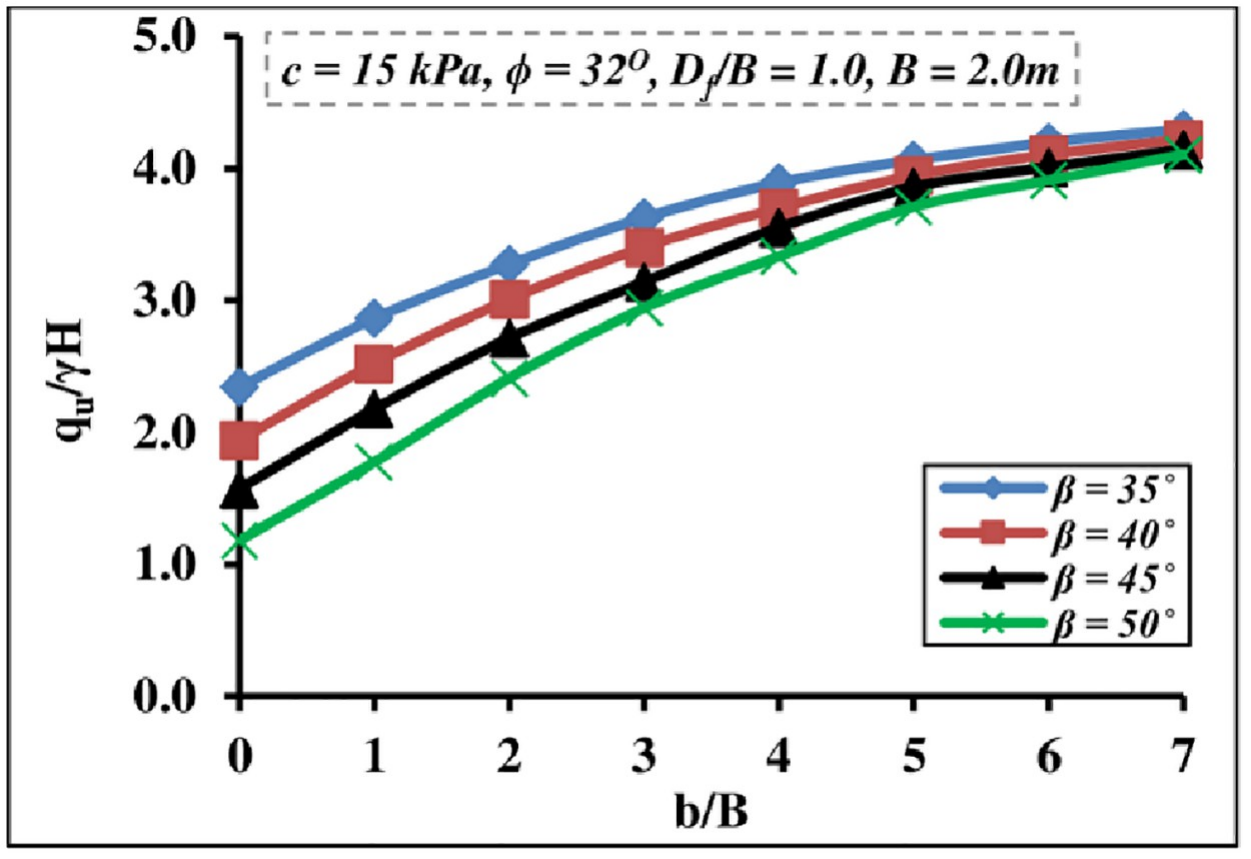
Combined influence of *β* and *b/B* ratio on *q*_*u*_*/γH*.

The effect of the foundation position *b/B* from the slope edge and the slope inclination *β* on the *q*_*u*_/*γH* of strip foundation built on the earthen slope crest is depicted in Fig 8. This graph illustrates that as the slope inclination increases, so does the BC. This is due to the free flow of dirt on the slope surface outward and a reduction in soil confinement or passive resistance from the side slope, which results in a decrease in footing bearing pressure. The outcomes show that the *q*_*u*_/*γ H* value is strongly related to the *b/B* ratio up to a critical ratio. Hence, at a small setback distance ratio, slope instability increases, soil confinement, and passive resistance decrease, and the footing-soil system stiffness is adversely affected, resulting in a drop in bearing pressure. The slope angle impact fades away at around *b/B* = 6, and the *q*_*u*_/*γ H* does not vary significantly for the further *b/B* ratio. This conclusion supports the findings arrived at by [52, 53]. Furthermore, the *q*_*u*_/*γ H* improvement rate is greater on steep gradient slopes than on low (gentle) gradient slopes.

#### 4.1.3 Effect of soil cohesion

**Fig 9:**
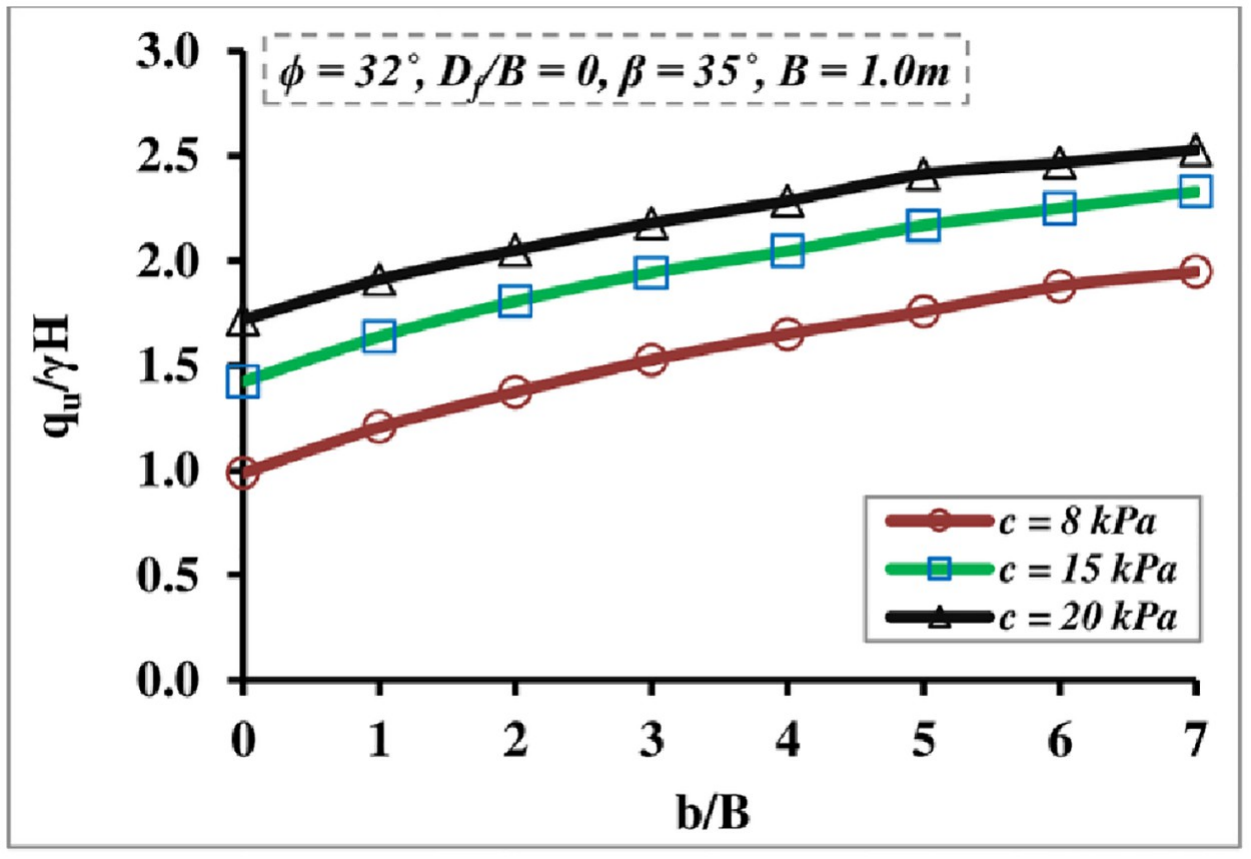
Impact of *c* and *b/B* ratio on *q*_*u*_*/γH*.

Fig 9 highlights the combined impact of the soil cohesion c variation and the *b/B* ratio on the BC; it shows that both have considerable influence on the *q*_*u*_/*γ H* value. It depicts that the *c* and *b/B* ratios positively correlate with the *q*_*u*_/*γ H* magnitude, the enhancement in the *q*_*u*_/*γ H* becoming insignificant after *b/B* = 6, and the impact being more tangible at larger c values. Improvement of *q*_*u*_/*γ H* satisfies the reality that increases in soil cohesion include improvements in the shear resistance of the foundation soil.

#### 4.1.4 Soil friction angle impact

**Fig 10:**
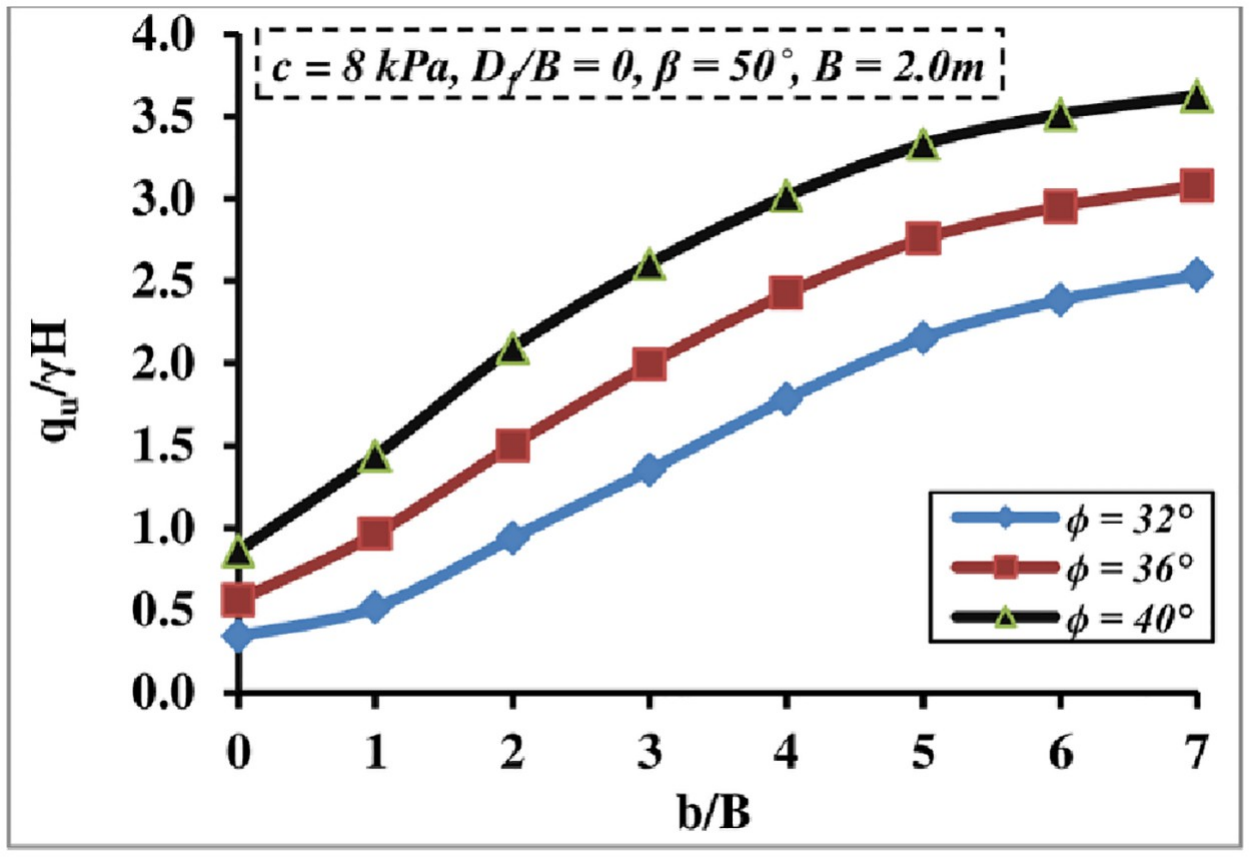
Impact of *ϕ* and *b/B* ratio on *q*_*u*_*/γH*.

Fig 10 depicts the combined effect of the friction angle *ϕ* and *b/B* ratio on *q*_*u*_/*γ H* value; both significantly impact the BC. It is claimed that the *ϕ* and *b/B* ratio have a proportionate relationship with the BC amount, the increase in the *q*_*u*_/*γ H* being inefficient after *b/B* = 6, and the impact became more pronounced at higher *ϕ* values. The increase in *q*_*u*_/*γ H* confirms the changes in soil friction angle and increases foundation soil shear resistance.

#### 4.1.5 Embedment depth impact

**Fig 11:**
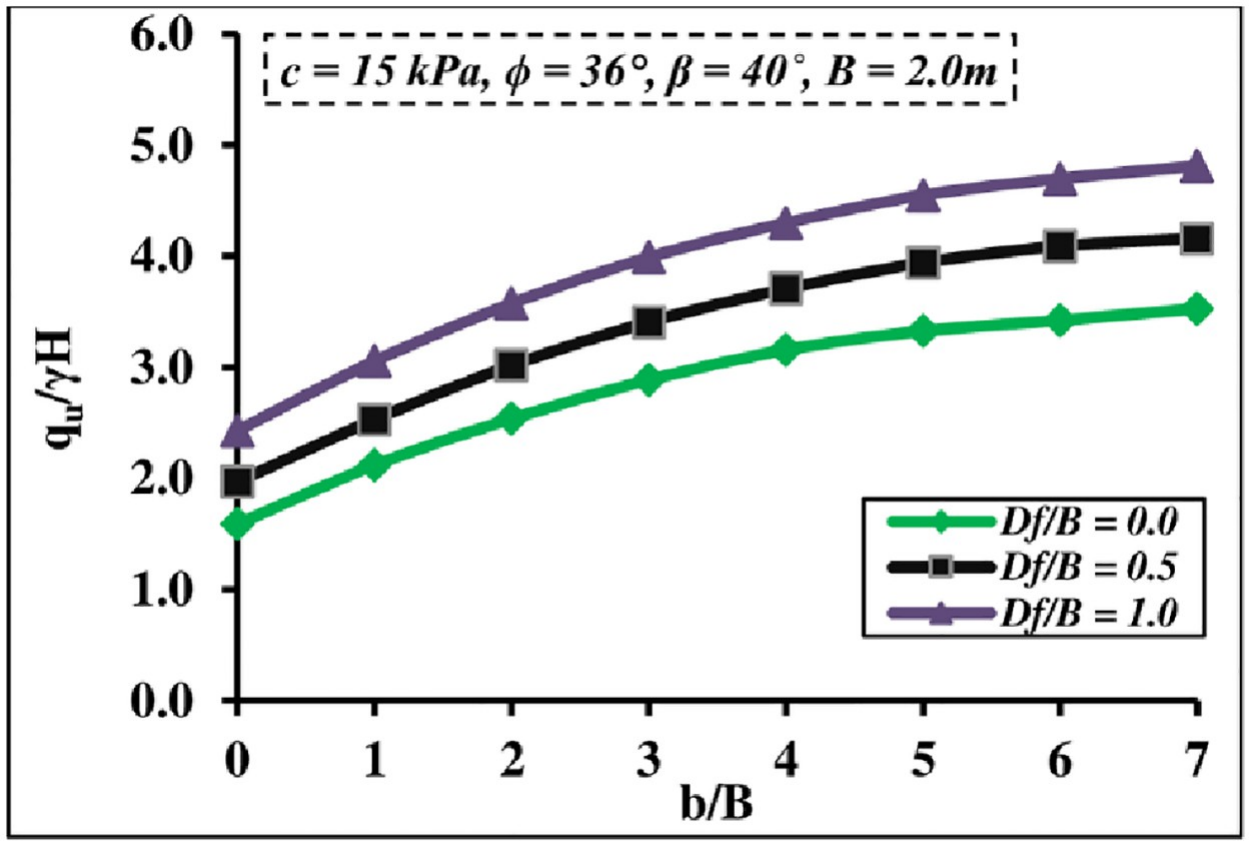
Effect of *D*_*f*_ and *b/B* ratios on *q*_*u*_*/γH*.

Fig 11 depicts the combined effect of the footing embedment depth *D*_*f*_*/B* and the *b/B* ratios, both of which have a significant effect on the *q*_*u*_/*γ H* value. It is seen that increasing of *D*_*f*_*/B* and *b/B* ratios increases the *q*_*u*_/*γ H* value. This is due to rising soil confinement, which raises the passive resistance zone. Furthermore, increasing *q*_*u*_/*γ H* value becomes invaluable after *b/B* = 6 and has a greater influence at a higher *D*_*f*_*/B* ratio.

#### 4.1.6 Failure mechanism

**Fig 12:**
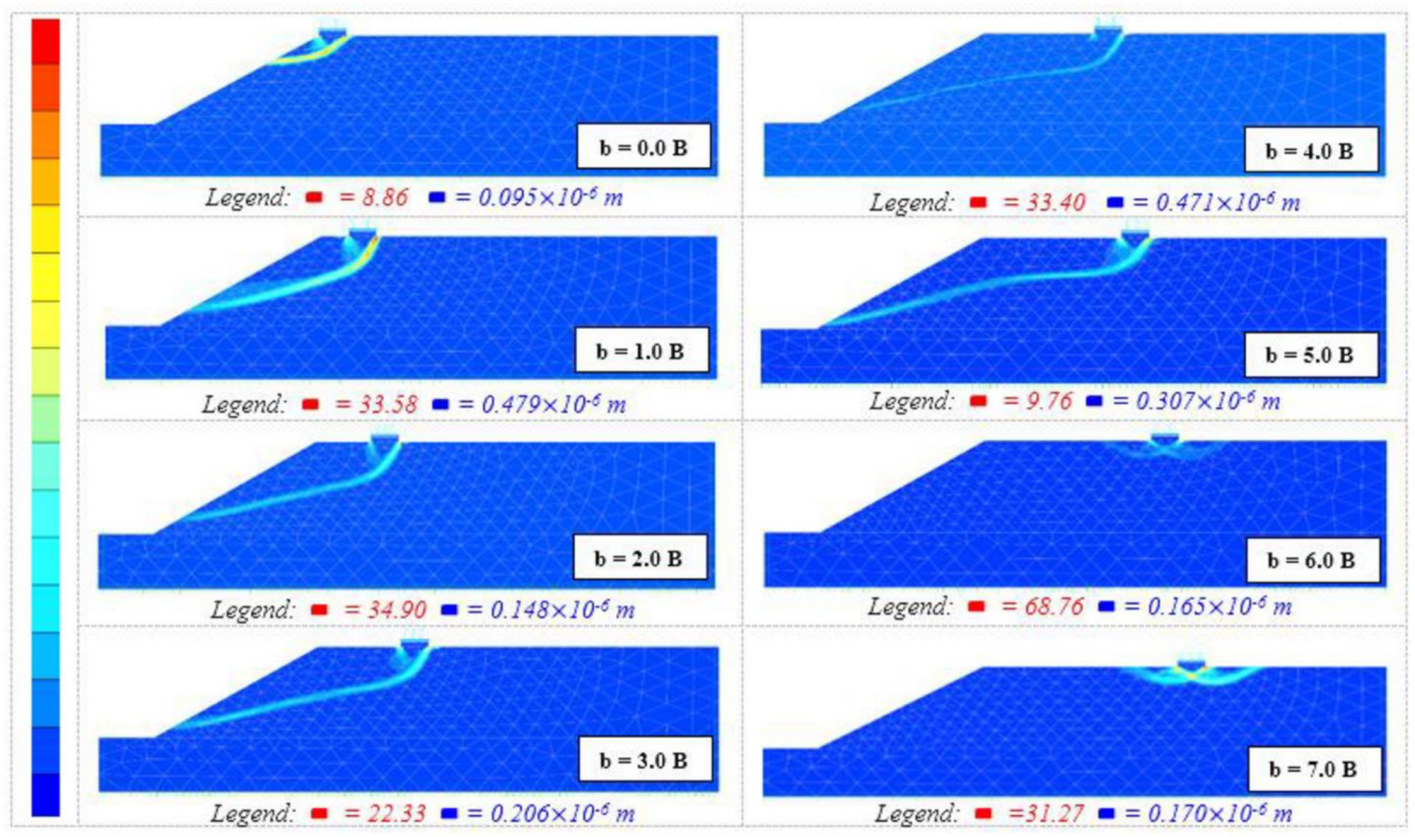
Failure pattern generated under foundation with *b/B* ratio. (*B = 2m; D*_*f*_ *= 0*.*0 B; ϕ = 40°; c = 8; β = 40°*).

In the current research, the soil failure pattern generated beneath a strip foundation placed on the slope crest has been analyzed up to *b/B* = 7 for various *β* and *b/B* ratios to determine the key *b/B* ratio. Then the impact of the *β* fades away. Fig 12 displays how the slope affects the passive zone formed under the foundation and that the failure pattern is one-sided only and toward the slope direction up to *b/B* = 6, affecting the soil bearing pressure and overall slope stability. The failure mechanism established after *b/B* = 6 is analogous to the failure mechanism developed on level ground or flat topography.

### 4.2 Numerical results of double-strip foundations

Table 4 shows the parameters used with the aid of the model displayed in Fig 3 to evaluate the interfering impact of two strip foundations lying on the earthen slope crest on the (*q*_*u*_/*γ H*) value. The statistical characteristics of input and target data are presented in Table 5.

**Table 4:** The considered parameters in the numerical model of double strip footings.

			*q*_*u*_ */γH*			
*β°*	*B (m)*	*D*_*f*_ */B*	*c (kPa)*	*ϕ°*	*b/B*	*S/B*
35						1.0
40	1.0	0.0	8	32	0.0	2.0
45	2.0	1.0	15	36	2.0	3.0
50			20	40		4.0
						5.0

**Table 5:** Statistical analysis of input and target data used in the EPR model for double strip foundations.

	Minimum	Maximum	Mean	Median	Std. deviation
*B (m)*	1	2	1.5	1.5	0.500
*c (kPa)*	8	20	14.333	15	4.923
*ϕ°*	32	40	36	36	3.267
*D*_*f*_ */B*	0	1	0.5	0.5	0.500
*β°*	35	50	42.5	42.5	5.592
*b/B*	0	2	1	1	1.000
*S/B*	1	5	3	3	1.415
*q*_*u*_ */γH*	0.273	4.771	1.916	1.803	0.838

#### 4.2.1 Effect of foundation width

**Fig 13:**
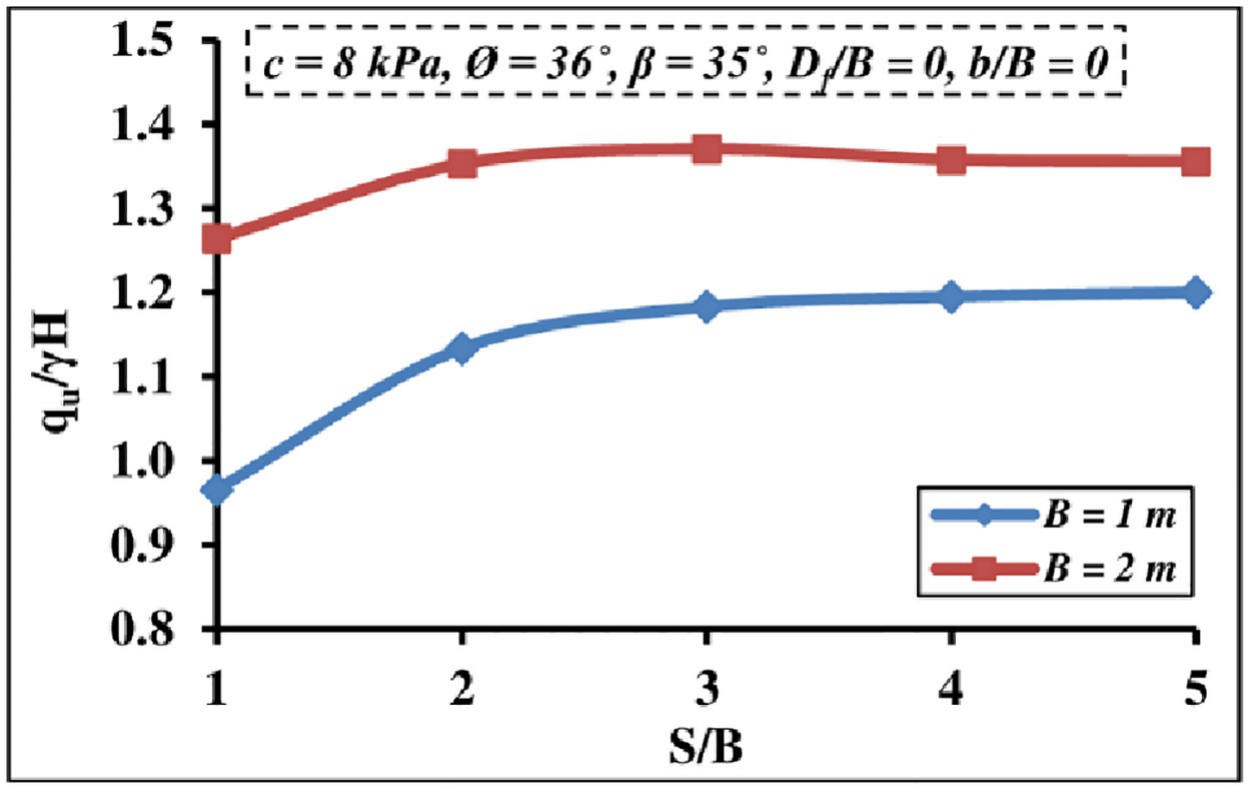
Interference impact of different *B* on *q*_*u*_*/γH*.

The impact of footing widths on the *q*_*u*_/*γ H* magnitude of two closely stripped foundations is shown in Fig 13. The outputs indicated that the *q*_*u*_/*γ H* value increases with the foundation width. This referred to the reality that the stress zone and failure patterns extend to a wider area and a deeper depth due to the wider foundation base, as a result, the bearing pressure improves. Furthermore, it is depicted that as the *S/B* ratio increases until it reaches 3–4, the interfering effect disappears completely.

#### 4.2.2 Impact of soil cohesion

**Fig 14:**
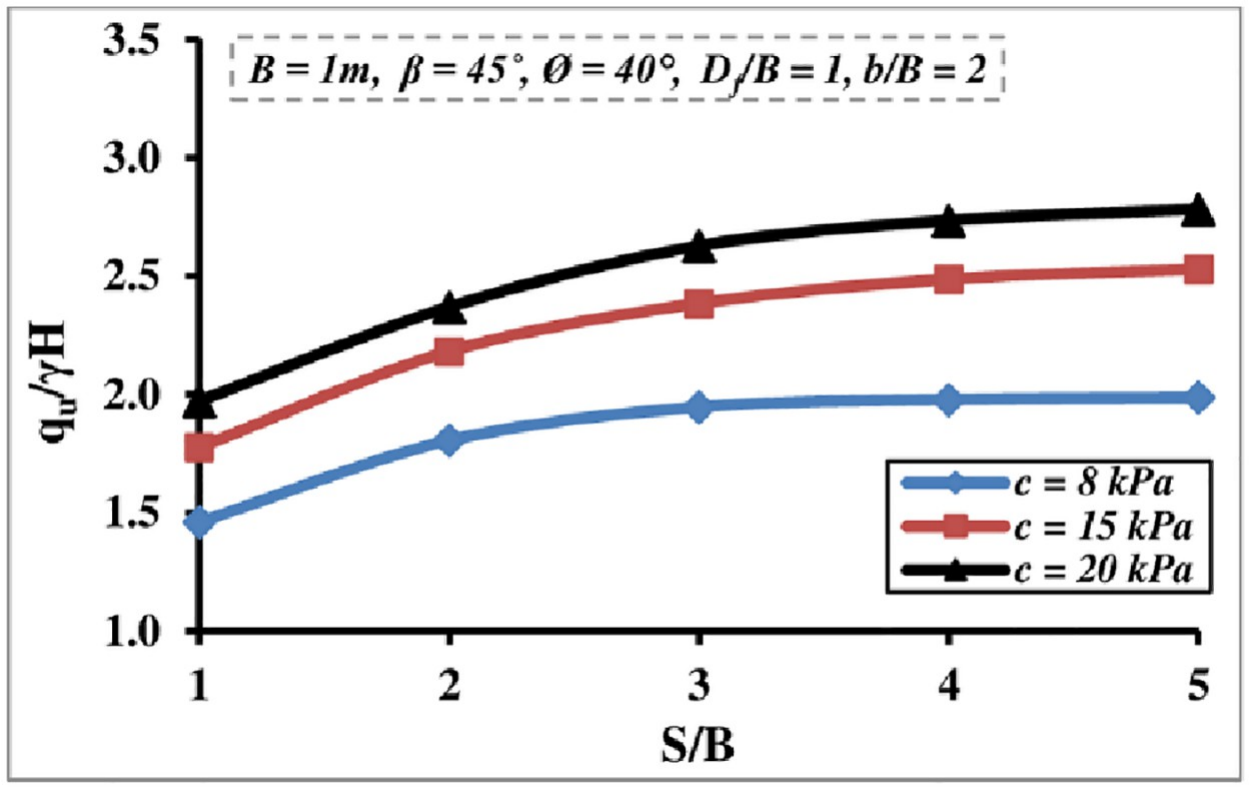
Interference effect of cohesion of soil on *q*_*u*_*/γH*.

Fig 14 displays the effect of varying foundation soil cohesion levels on interfering strip footing *q*_*u*_/*γ H* value. As seen, as the foundation soil cohesiveness increases the *q*_*u*_/*γ H* magnitude increases until the *S/B* = 3–4, at which point the interfering impact diminishes and the footings act independently. This is because the soil resistance against soil shear failure has increased.

#### 4.2.3 Impact of soil friction angle

**Fig 15:**
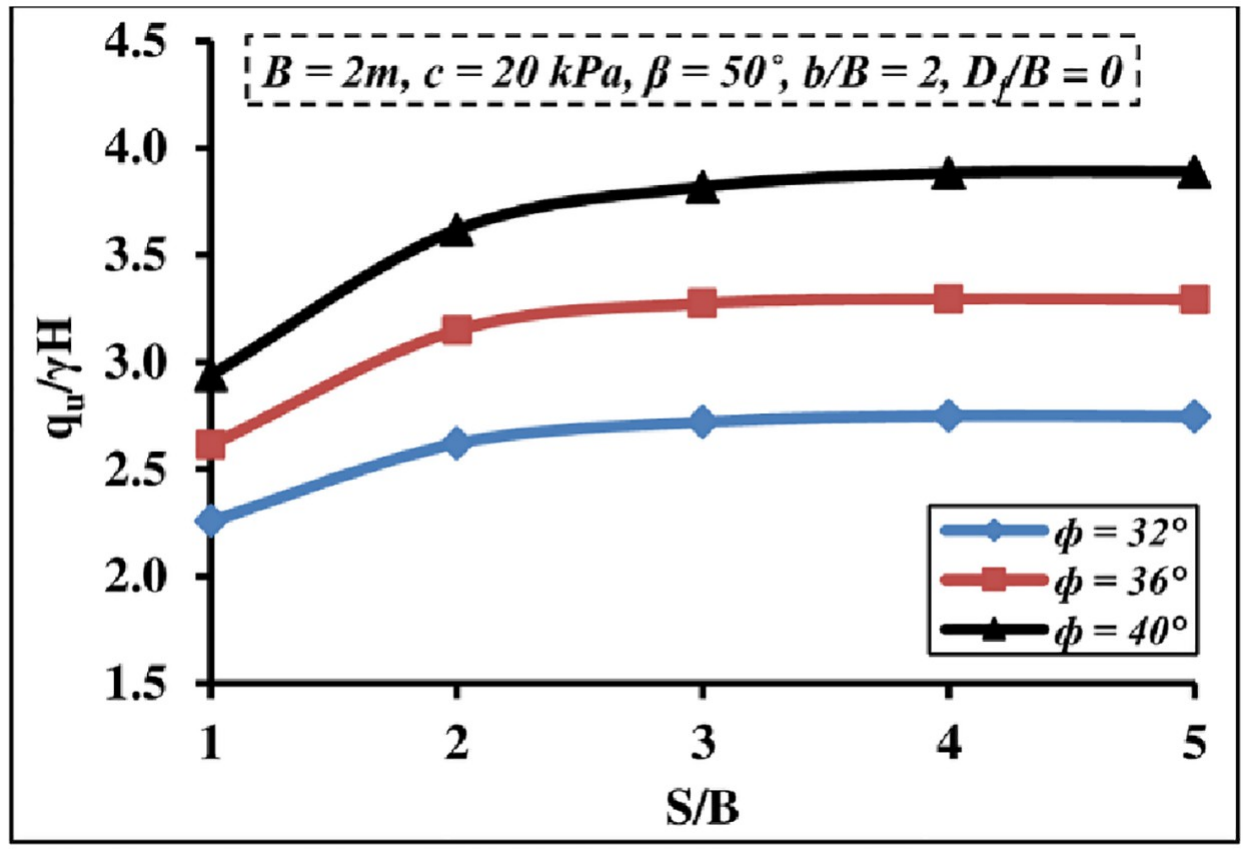
Interference impact of several *ϕ* of foundation soil on *q*_*u*_*/γH*.

Fig 15 demonstrates the impact of varying *ϕ* values of the foundation soil on the *q*_*u*_/*γ H* value of interfering strip foundations. It is reported that the *q*_*u*_/*γ H* value increased as *ϕ* values of the foundation soil increased; however, the interfering effect vanished once the *S/B* ratio reached 3–4. The higher *ϕ* values, the greater the foundation soil resistance, and hence the greater the load necessary to cause the soil collapse.

#### 4.2.4 Effect of embedment depth

**Fig 16:**
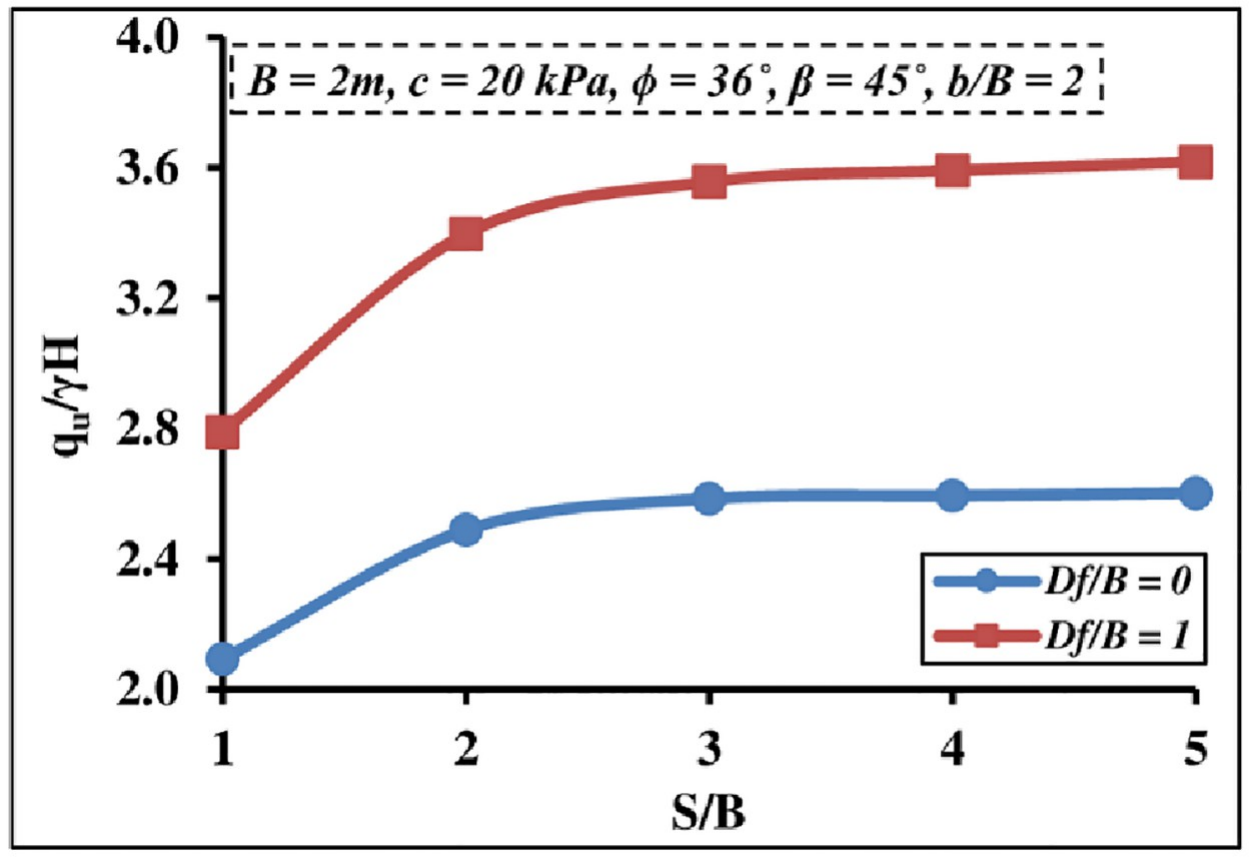
Interference influence of different *D*_*f*_*/B* ratios on *q*_*u*_*/γH*.

Fig 16 depicts the effect of the *D*_*f*_*/B* ratio on a two-closely strip foundation. It is noticed that the *D*_*f*_*/B* ratio has a remarkable impact on the *q*_*u*_/*γ H* and that its value increases as the *D*_*f*_*/B* ratio increases. This is due to the increasing soil confinement, which produces an increase in the passive resistance area beneath and around the foundation. Also, the results showed that when *q*_*u*_/*γ H* increases, the *S/B* ratio increases too until it reaches 3–4 and then the interfering impact disappears altogether.

#### 4.2.5 Impact of setback distance, slope angle, and spacing

**Fig 17:**
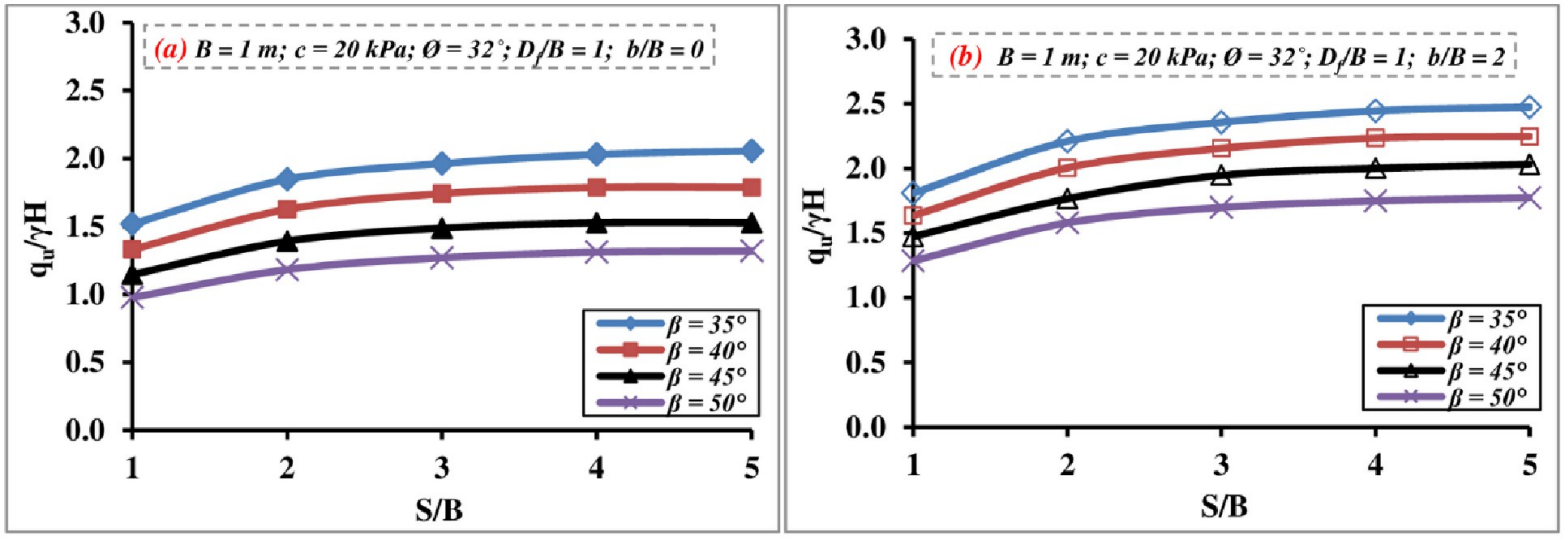
Interference effect of various ratios of *b/B* and *β* on *q*_*u*_*/γH*.

Fig 17 shows the effect of the spacing *S/B* ratio on the *q*_*u*_/*γ H* value of two continuous foundations located over the slope crest for various *β* and *b/B* ratios. It is observed from the outcomes that the *q*_*u*_/*γ H* value is in most cases, less than that of a single-case footing under the same conditions. Then it gradually rises to *S/B* = 3–4 to reach or sometimes exceed the value of the single strip foundation, after which it roughly rests constant and the interference influence gradually vanishes and the footings behave as individual ones. This conclusion supports that reached by [11, 46], who stated that the interference of two strip footings acts up to an *S/B* = 3 ratio, after which its impact fades.

#### 4.2.6 Failure mechanism

**Fig 18:**
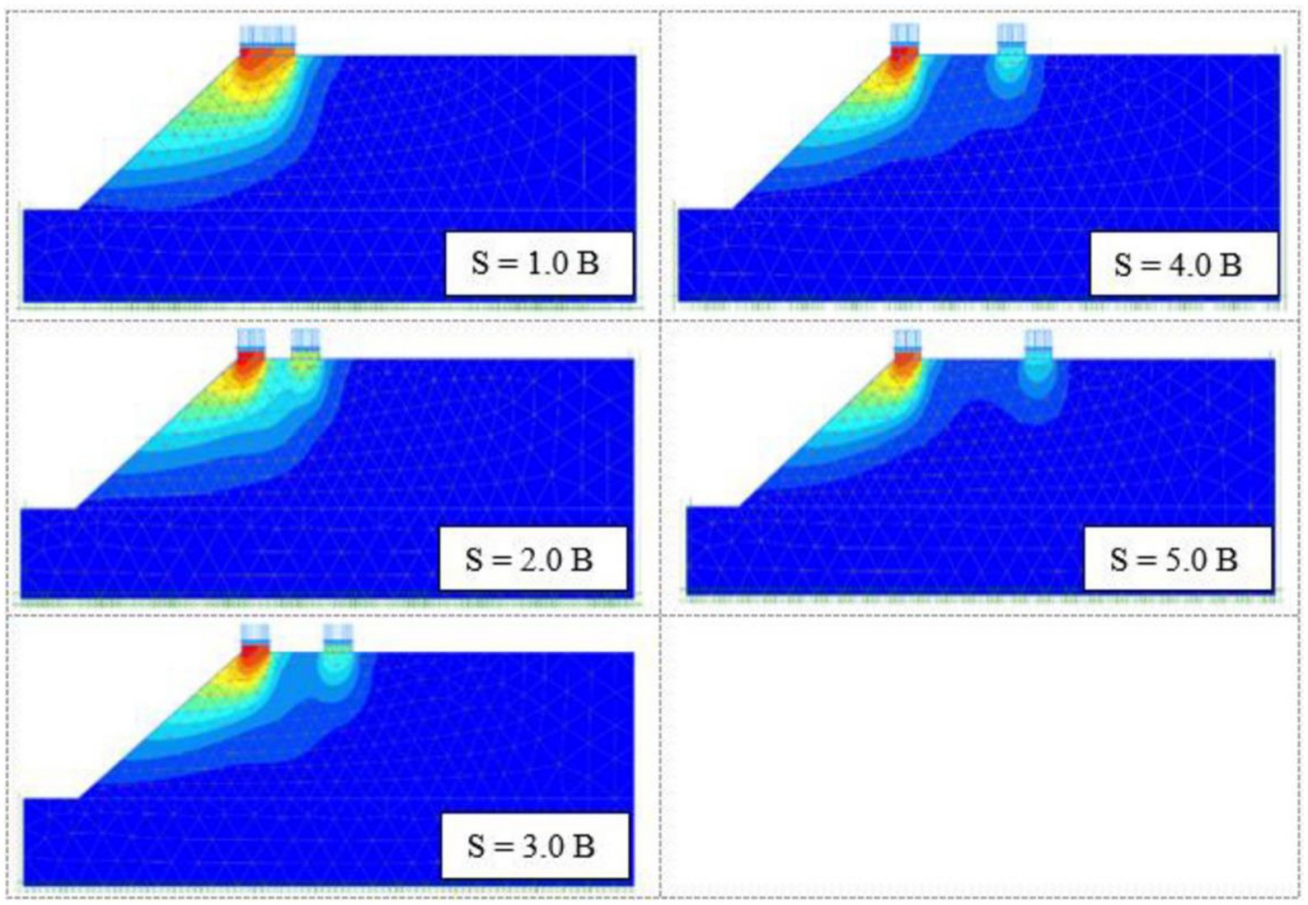
Failure pattern generated under two foundations with different *S/B* ratios. (*B = 2m*, *c = 8*, *ϕ = 40°*, *D*_*f*_*/B = 0*, *β = 40°*, *and b/B = 0*).

The soil failure pattern generated beneath two strip foundations on the slope crest has been tested up to *S/B* = 5 for several *β* and *S/B* ratios to determine the critical *S/B* ratio and the impact of the β. Fig 18 displays that the presence of the slope greatly impacts the passive region formed below the foundation and that the failure pattern is one-sided only and toward the slope surface up to about *S/B* = 3–4, and consequently overall slope stability and the bearing pressure. After that, the developed failure zone is separated, and the second foundation does not affect the performance of the first one.

### 4.3 The EPR mathematical models

One of the superior EPR-MOGA features is its capability to generate many models for a particular physical problem, providing the modeler with the resilience to select the best expression from among the developed expressions depending on parametric study or engineering judgment. However, before beginning the EPR operation, some variables need to be modified to regulate the modeling architecture development process. These variables can be utilized to influence the optimization approach employed, such as the exponent ranges, the desired terms number in the mathematical model(s), mathematical structures, and the function types to generate the models. To run EPR-MOGA, besides the clear training dataset, two distinct parameter sets that control the linear regression steps and the evolutionary procedure should be appointed: the general structure and the terms number of the model, the polynomial exponents range, the type of regression, the estimation method coefficients, and the optimization strategy based on the Pareto dominance criteria [39, 54] and at the end of the modeling phase a set of model solutions is produced. A reasonable setting of such parameters positively affects the procedure’s run time.

In this case, the model with the fewest terms (to increase simplification and decrease complexity) and the highest coefficient of determination (COD = R^2^) value (to ensure maximum possible fitness) will be chosen. The total dataset is divided randomly into three sets: 70% for training, 15% for testing, and 15% of the invisible data in both the training and testing processes to validate the predicted EPR model. The trial-and-error technique is conducted to get the most effective (optimal) EPR model, depending on the R^2^ value. Table 6 describes the parameters used to construct the EPR model.

**Table 6:** EPR setting parameters.

Parameter	EPR tuning
Expression structure	Y = sum (*a*_i_*X_1_*X_2_*f(X_1_)*f(X_2_)) + *a*_0_
Function type	No function
Terms	6
Exponents range	0; 0.5; 1; 1.5; 2;– 0.5;– 1;– 1.5;– 2
GA	15
Bias (*a*_0_)	yes
Regression method	Least Square

#### 4.3.1 Model assessment for single-strip foundation

**Fig 19:**
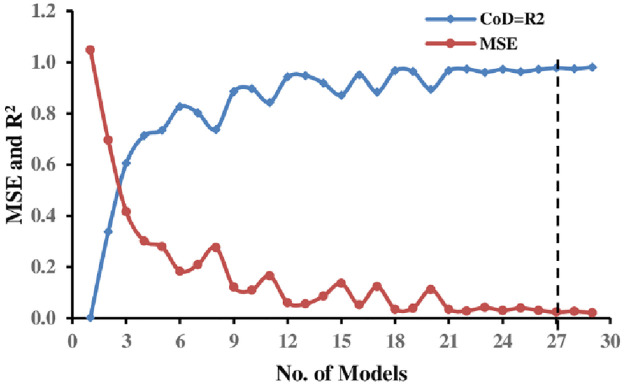
Developed EPR models with MSE and R^2^ (single strip foundation).

The EPR approach generates several mathematical expressions to calculate the normalized bearing capacity *q*_*u*_/*γH* (corresponding to *5% B* settlement) of a strip foundation situated on the earth slope crest. Factors considered include; *B*, *c*, *ϕ*, *D*_*f*_*/B*, *β*, and *b/B*. Eq (13) summarizes the best (optimal) EPR model No. 27 in Fig 19 selected among many constructed models according to the previously mentioned statistical criteria in this study that represents the particular geotechnical engineering issue under consideration:

$$\begin{array}{c} \frac{q_{u}}{\gamma \;H}=9.9392\times 10^{-2}\;\left(\frac{b}{B}\right)-5.9459\times 10^{-2}\;\beta +0.1174\;\varphi +0.4116\;c^{0.5}\\ +0.5311\;B\;\left(\frac{D_{f}}{B}\right)+5.5364\times 10^{-3}\;\beta \;B^{1.5}\;{\left(\frac{b}{B}\right)}^{0.5}-2.0183 \end{array}$$ (13)

The generated model provided in Eq (13) was evaluated by estimating the accuracy performance using the five indices presented in Eqs (8)–(12) and comparing it to its optimal value to estimate overall error and overall accuracy. The statistical performance indicators of the proposed EPR-MOGA model (Eq 13) are presented in Table 7. The table indicates the model’s strength because the MAE and RMSE values obtained are very close to zero. This is also confirmed by the obtained R^2^ values (0.978, 0.974, and 0.977) and variance accounting for VAF (97.8, 97.4, and 97.7) for training, testing, and validation, respectively. In addition, according to A^**15**^-index values, it is also obvious that 93.4%, 94.2%, and 91.9% of the prediction values are within an error range of ±15% compared to measured values, which implies a very good prediction overall.

**Table 7:** Performance indicator values for the EPR model in (Eq 13).

Phase	R^2^	MAE	RMSE	VAF %	A^15^-Index
Training	0.978	0.0031	0.153	97.8	0.934
Testing	0.974	0.0026	0.155	97.4	0.942
Validation	0.977	0.0004	0.162	97.7	0.919

Perfect R^2^ = 1; Perfect MAE = 0; Perfect RMSE = 0; Perfect VAF = 100%; Perfect A^15^-index = 1

**Fig 20:**
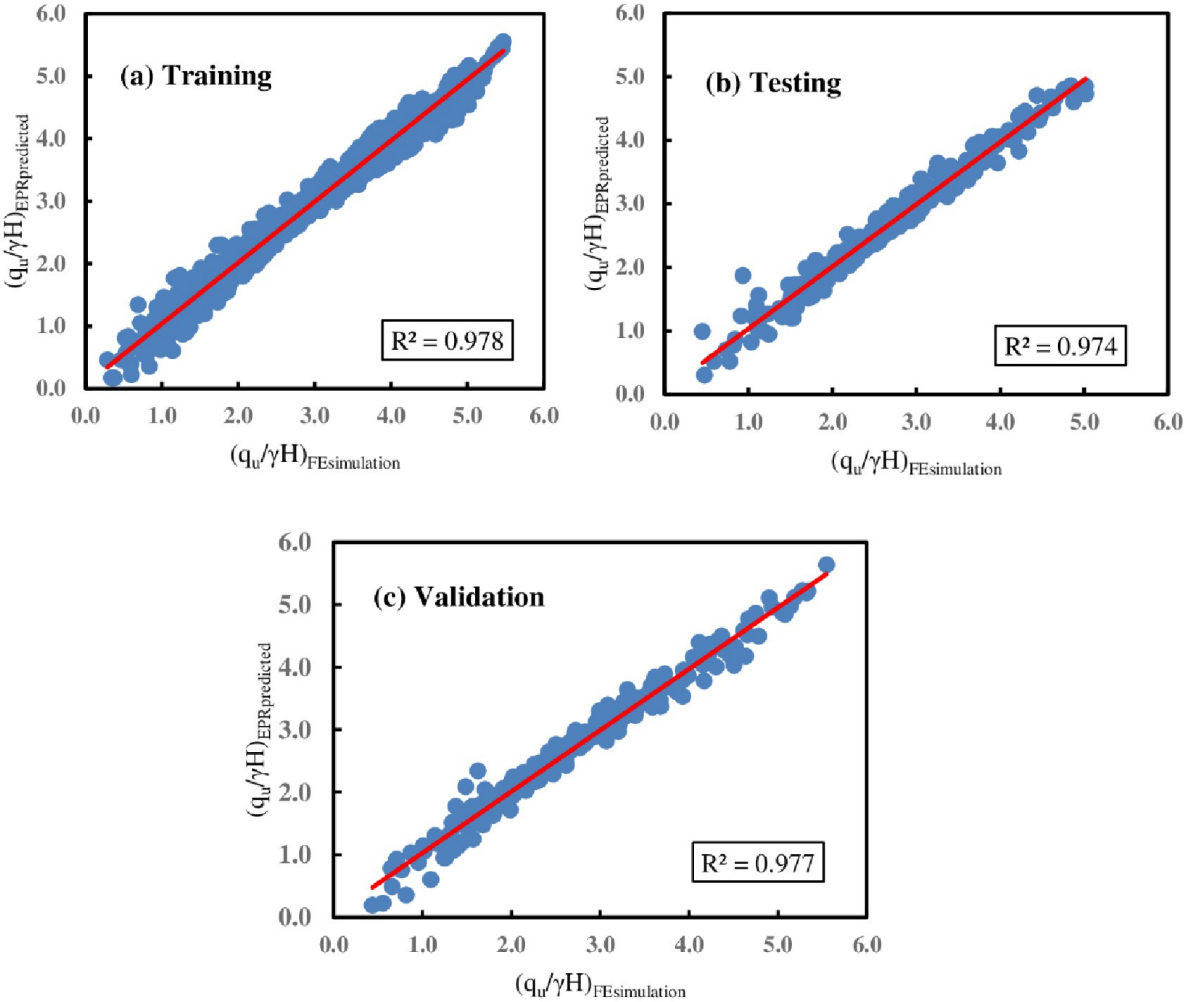
Results comparison obtained by FE and EPR for a single-strip foundation.

**Fig 21:**
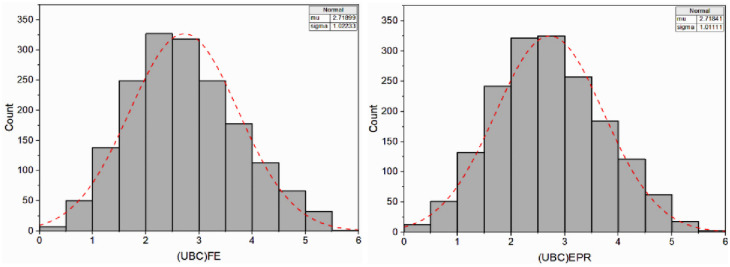
Distribution histograms of FE simulated and EPR predicted bearing capacities.

Fig 20 displays the comparison of outcomes determined using FE simulation with those predicted by EPR for the training, testing, and validation phases, respectively, with their corresponding correlation factors. As can be seen from the figure, the FE simulation results and the EPR predictions show a strong relationship. Furthermore, Fig 21 illustrates the histograms for the FE and EPR ultimate bearing capacities.

#### 4.3.2 Model assessment for double-strip foundations

**Fig 22:**
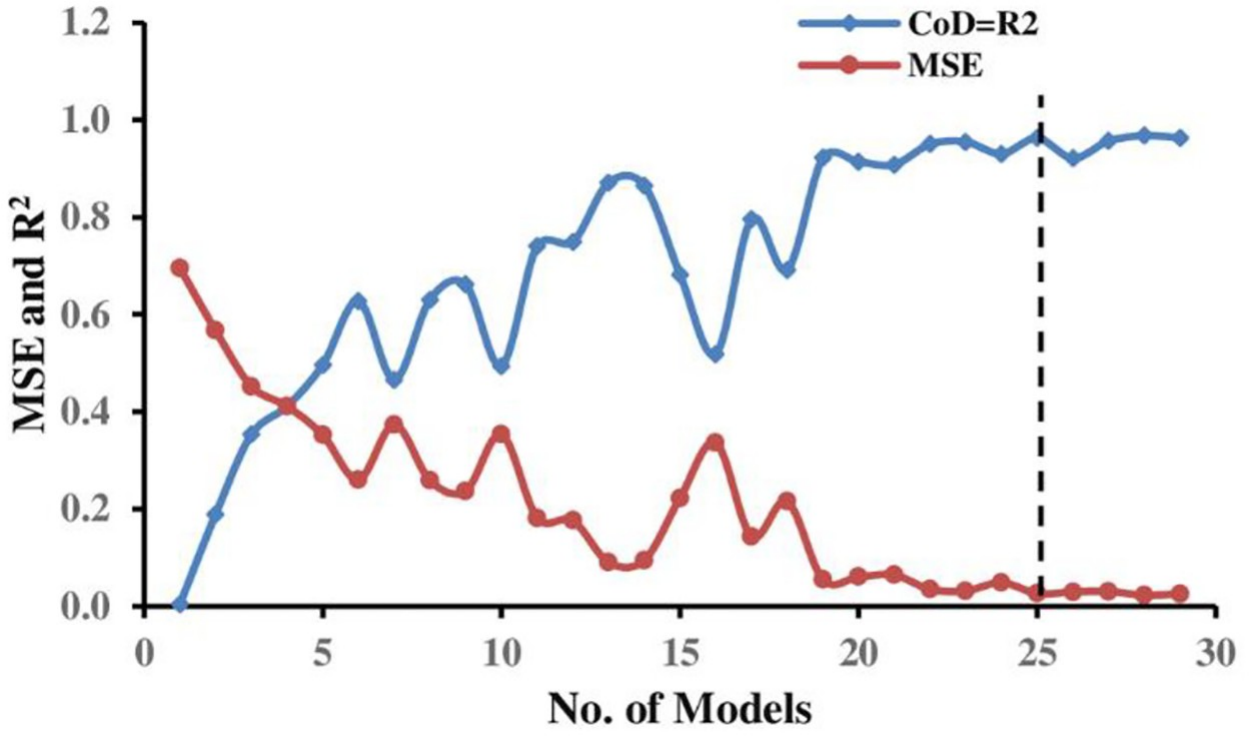
Developed EPR models with MSE and R^2^ (double-strip foundations).

In this case, the most suitable EPR expression considered to predict the normalized bearing capacity *q*_*u*_/*γ H* (corresponding to *5% B* settlement) of two strip foundations resting on the earth slope crest while accounting for various parameters such as *B*, *c*, *ϕ*, *D*_*f*_*/B*, *β*, *b/B*, and *S/B*, is explained in Eq (14) and illustrated as model No.25 in Fig 22.


$$\begin{array}{c} \frac{q_{u}}{\gamma \;H}=1.048\times 10^{-2}\;\varphi^{1.5}-\frac{0.4997}{{\left(\frac{S}{B}\right)}^{1.5}}-5.5477\times 10^{-3}\beta^{1.5}+0.4196\;c^{0.5}\\ +0.3198\;B^{1.5}\;{\left(\frac{D_{f}}{B}\right)}^{0.5}+0.2722\;B^{1.5}\;{\left(\frac{b}{B}\right)}^{0.5}-0.8652 \end{array}$$ (14)

Similarly, the accuracy performance of the proposed model provided in Eq (14) was evaluated using the five indices shown in Eqs (8)–(12) and comparing the results to the optimum value of each indicator to measure overall error and accuracy. Table 8 displays the statistical performance metrics of the developed EPR-MOGA model (Eq 14). The table illustrates that the constructed model has high accuracy, as the obtained MAE and RMSE values are close to zero. This is corroborated by R^2^ values of 0.964, 0.960, and 0.958 for training, testing, and validation, respectively. Based on the performance indicators, it can be concluded that the proposed model is robust and can be used to quickly and easily analyze the BC of double-strip foundations located near the earth slope crest.

**Table 8:** Performance indicator values for the EPR model in (Eq 1^4^).

Phase	R^2^	MAE	RMSE	VAF %	A^15^-Index
Training	0.964	0.0005	0.159	96.4	0.884
Testing	0.960	0.0172	0.171	95.9	0.861
Validation	0.958	0.0086	0.174	95.8	0.849

Perfect R^2^ = 1; Perfect MAE = 0; Perfect RMSE = 0; Perfect VAF = 100%; Perfect A^15^-index = 1

**Fig 23:**
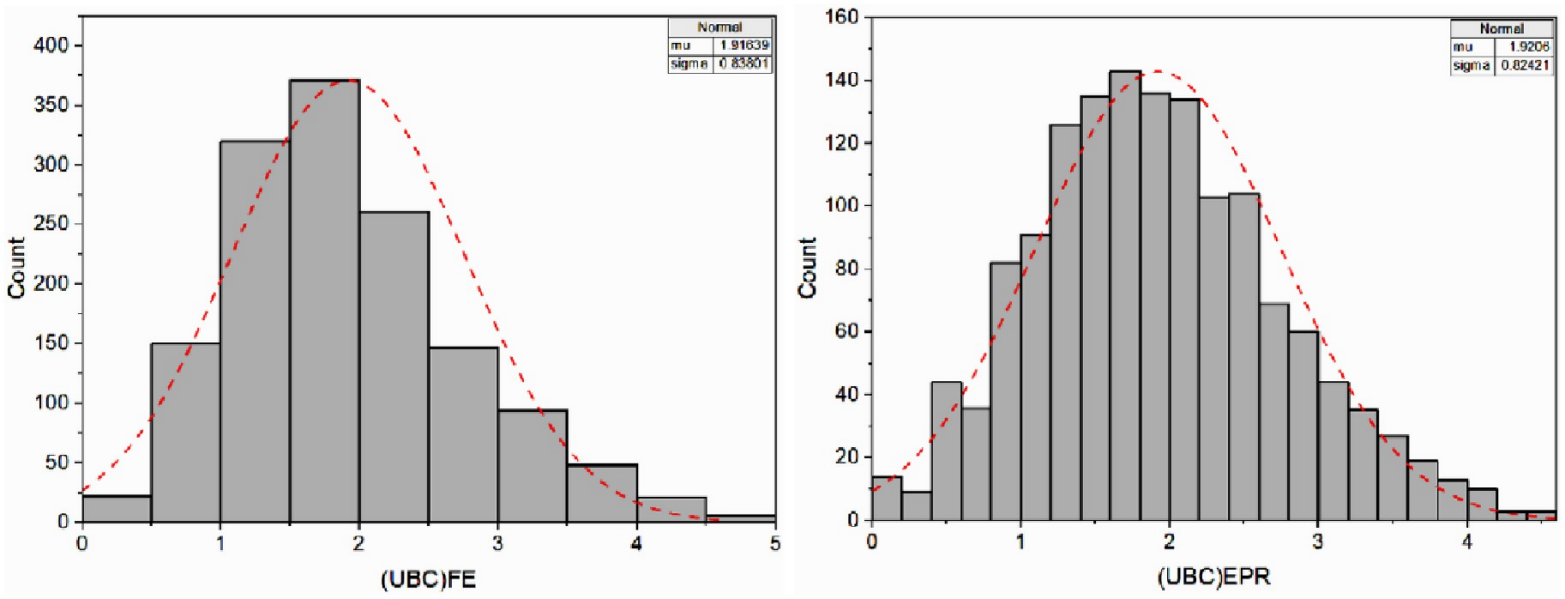
Distribution histograms of output predictions from FE and EPR models.

**Fig 24:**
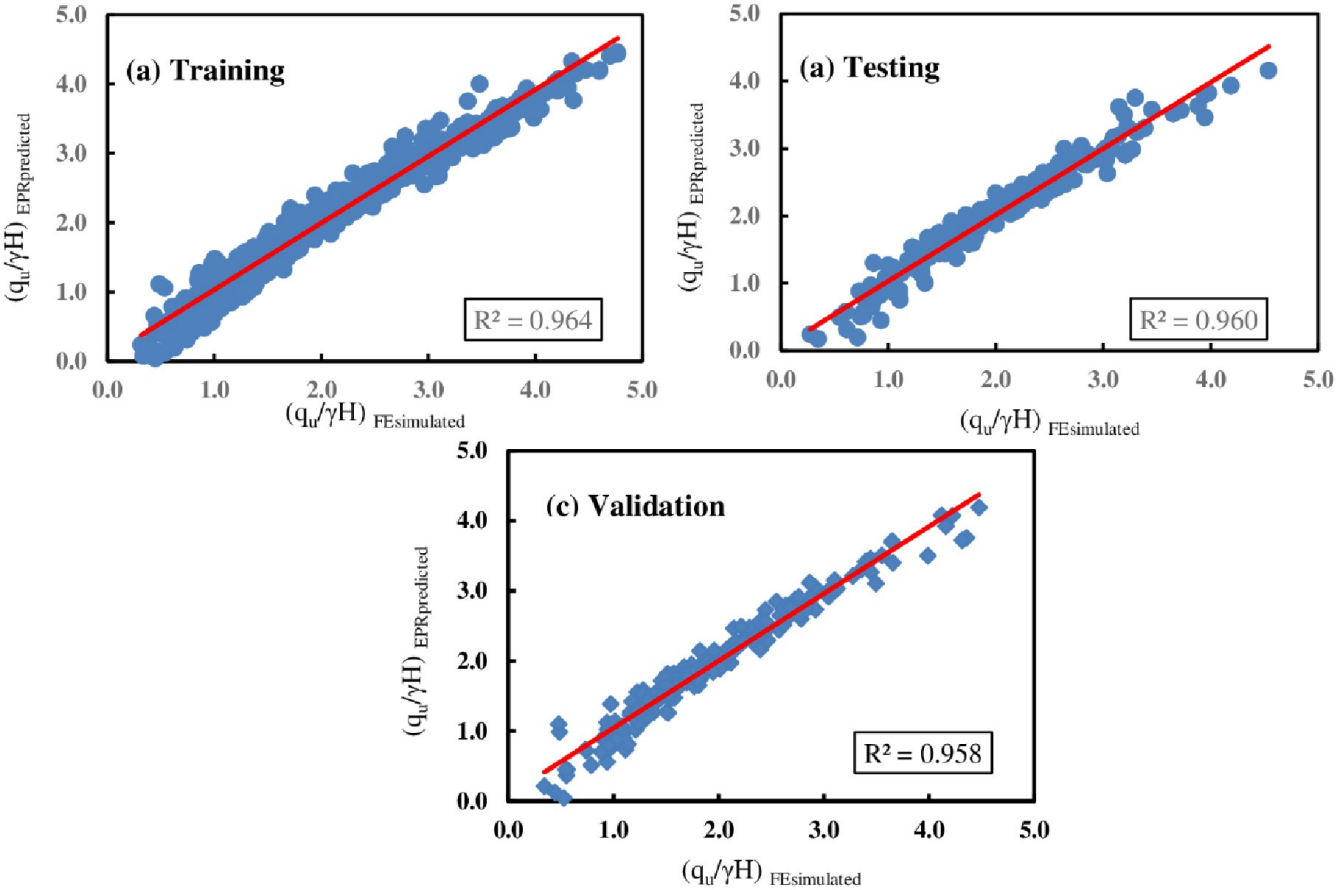
Results comparison obtained by FE and EPR for double foundations.

Fig 23 displays the histograms for the FE and EPR ultimate bearing capacities. Similarly, the comparison of results obtained using FE simulation and those predicted by the EPR equation for each of the training, testing, and validation processes is given in Fig 24 with its correlation factors, respectively. According to the figure, a notable correlation has been observed between the FE simulation results and the EPR predictions.

## 5. Sensitivity analysis of input variables

To analyze the impact of each independent (input) variable on the dependent (output) variable, a sensitivity analysis is needed. This analysis determines the range within which the model output could vary based on variations in the input parameters. Various approaches are available for this process in the literature, with the one-factor-at-a-time technique being a highly effective method [55]. As a result, it identifies the most sensitive input variable among all the other variables that can influence the outcomes of the model [56]. The method highlights how a single input parameter affects model outcomes, so it has been used in this study. In this method, the range of variation in each input variable is considered as the standard deviation above and below the mean value (i.e., *σ ± ȳ*). This is done by using the upper/lower bounds (UB and LB) of each input variable independently, while holding all other variables constant [57, 58]. The outcome of the sensitivity analysis carried out with the aid of the one-factor-at-a-time technique is highlighted in Table 9. It can be observed that footing width (*B)*, setback distance between the slope edge and foundation (*b/B)*, and soil internal friction *(ϕ°)* are more sensitive and play a dominant role in estimating the BC of individual and two interfering strip foundations set on a sloping crest.

**Table 9:** Rank of input variable’s importance from one-factor-at-a-time technique.

Input variable	Single strip foundation		Double strip foundations	
	UB & LB Range Diff.	Rank	UB & LB Range Diff.	Rank
*B (m)*	1.070	1	0.911	1
*c (kPa)*	0.544	5	0.554	6
*ϕ°*	0.767	3	0.616	3
*D*_*f*_ */B*	0.651	4	0.587	5
*β°*	- 0.452	6	-0.606	4
*b/B*	1.021	2	0.707	2
*S/B*			0.196	7

**Fig 25:**
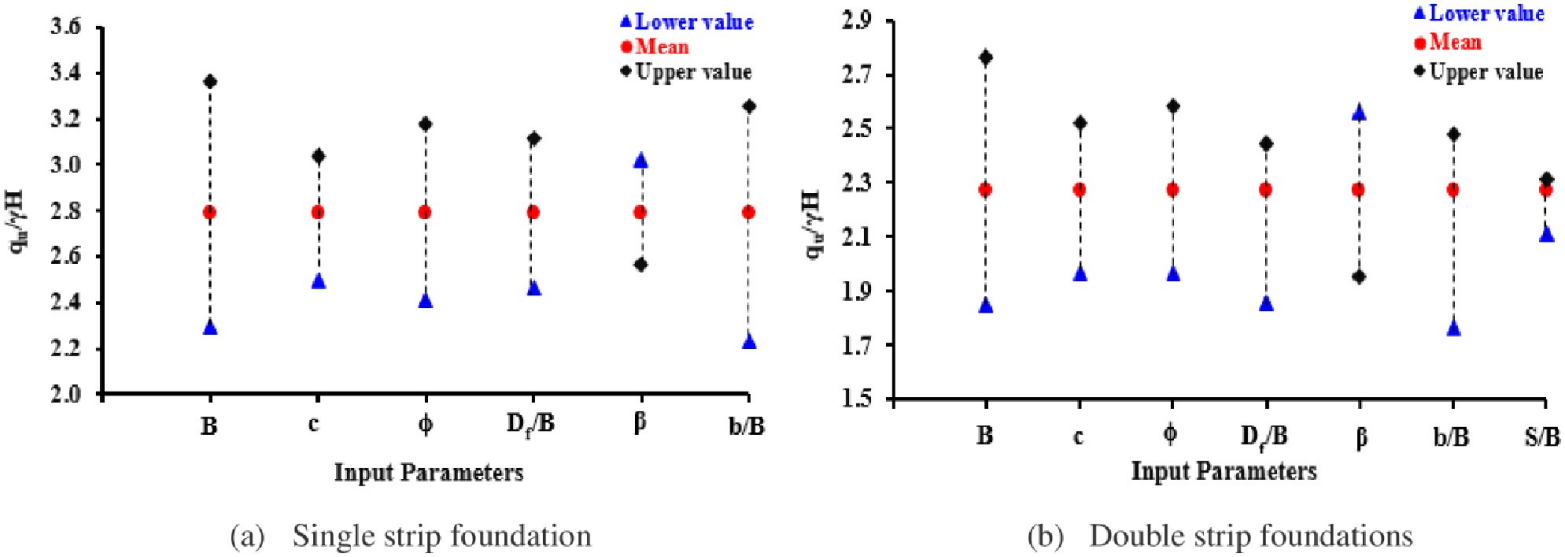
Input variable’s importance for the used dataset.

Fig 25 illustrates details of the input variable’s importance sensitivity analysis for both single and double-strip foundations. While their corresponding Pearson correlation matrices are shown in Table 10 which indicates that there is an excellent correlation (0.989 and 0.981) between simulated, (UBC)_FE_, and predicted, (UBC)_EPR_, results for single and double foundations respectively, showing that the developed models have noticeable capable to generate and predict the outcomes. Also, it depicts that the setback distance between the slope edge and foundation (*b/B)*, footing width (*B)*, and soil internal friction *(ϕ°)* have high correlation coefficients with the output and this coincides with results as previously obtained.

**Table 10:** Pearson’s correlation heatmap for input and target data.

***(a) Single strip foundations*.**									
	*B (m)*	*c (kPa)*	*ϕ°*	*D*_*f*_ */B*	*β°*	*b/B*	*(UBC)*_*FE*_	*(UBC)*_*EPR*_	
*B (m)*	1								
*c (kPa)*	0	1							
*ϕ°*	0	0	1						
*D*_*f*_ */B*	0	0	0	1					
*β°*	0	0	0	0	1				
*b/B*	0	0	0	0	0	1			
*(UBC)*_*FE*_	0.484	0.276	0.376	0.306	– 0.227	0.564	1		
*(UBC)*_*EPR*_	0.490	0.276	0.379	0.321	– 0.230	0.571	0.989	1	
***(b) Double strip foundations***									
	*B (m)*	*c (kPa)*	*ϕ°*	*D*_*f*_ */B*	*β°*	*b/B*	*S/B*	*(UBC)*_*FE*_	*(UBC)*_*EPR*_
*B (m)*	1								
*c (kPa)*	0	1							
*ϕ°*	0	0	1						
*D*_*f*_ */B*	0	0	0	1					
*β°*	0	0	0	0	1				
*b/B*	0	0	0	0	0	1			
*S/B*	0	0	0	0	0	0	1		
*(UBC)*_*FE*_	0.406	0.336	0.368	0.354	– 0.360	0.444	0.172	1	
*(UBC)*_*EPR*_	0.391	0.344	0.373	0.371	– 0.366	0.446	0.175	0.981	1

**Fig 26:**
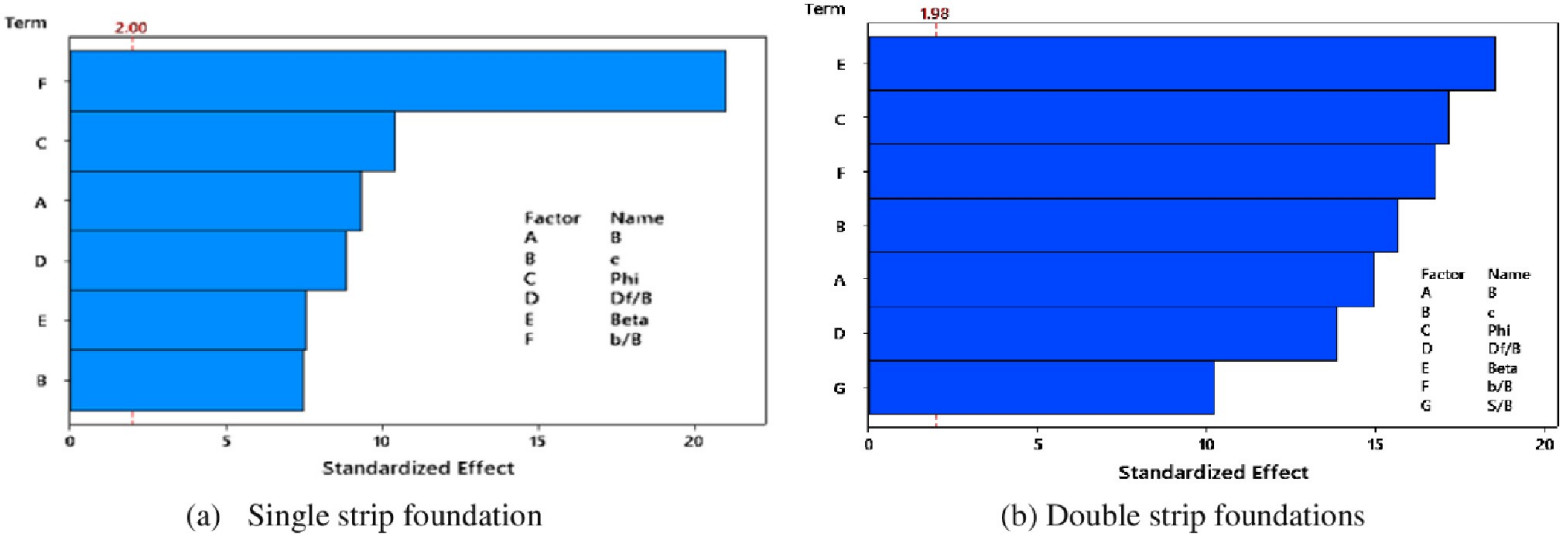
Input parameters interaction Pareto charts.

**Fig 27:**
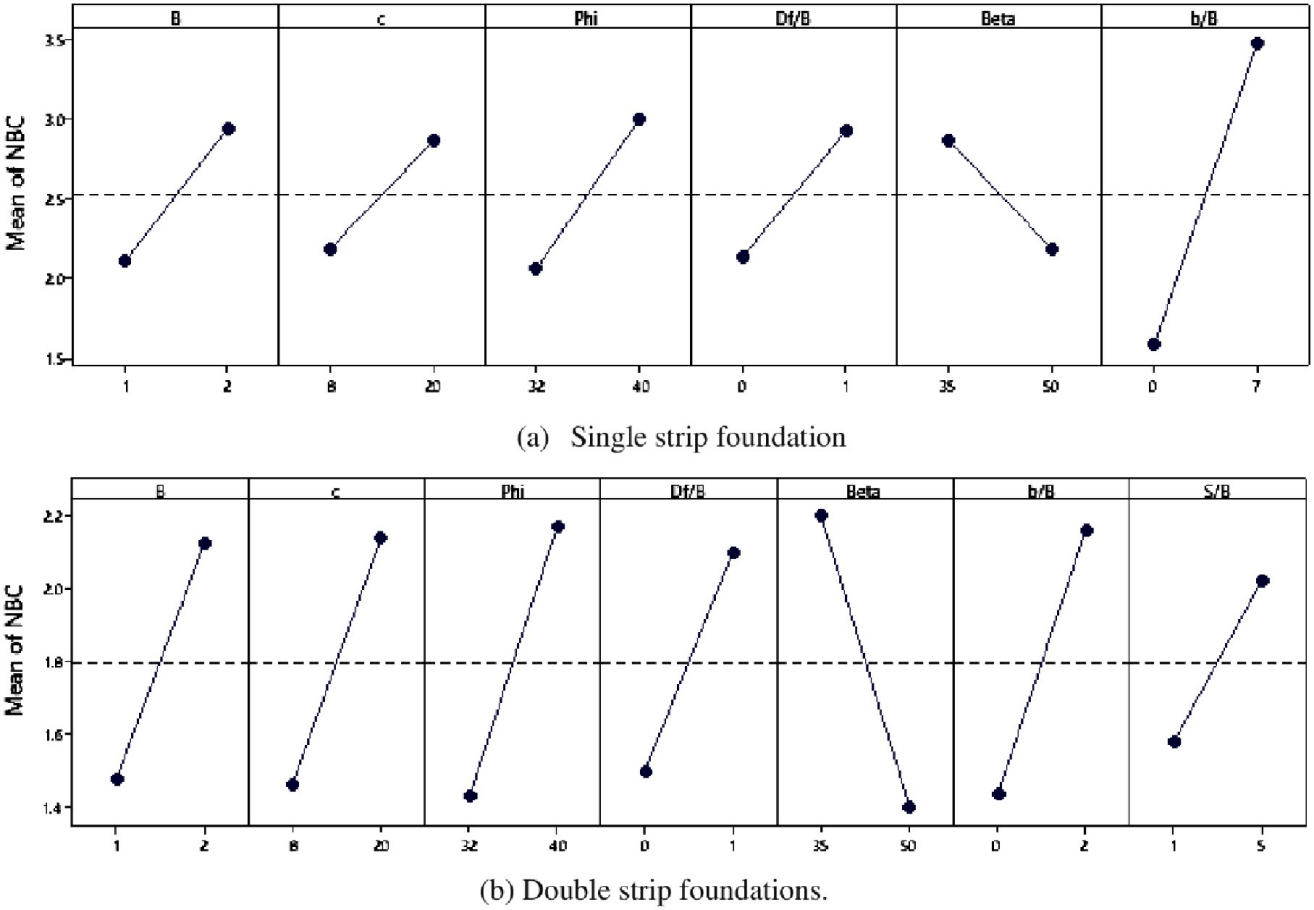
Main effects plot for NBC.

To confirm the sensitivity analysis outcomes performed by the one-factor-at-a-time technique, the factorial design approach was also conducted to capture the full complexity of the interactions between input variables. The results of the input parameter interactions for each of the single and double-strip foundations are presented in Pareto charts, as illustrated in Fig 26. All of the bars in each chart cross the reference line, indicating that all the used parameters are statistically significant. Depending on the results shown in Figs 26 and 27, the most effective parameters that influence the normalized bearing capacity (NBC) of a single strip foundation placed near an earthen slope are setback distance (*b/B*), soil friction (*ϕ*), and foundation width (*B*), while those in the case of the double-strip foundations are slope inclination (*β*), soil friction (*ϕ*), and setback distance (*b/B*). A comparison of two sensitivity analyses based on one-factor-at-a-time and factorial design shows that the optimal input parameters are the same (i.e., *b/B*, *ϕ*, and *B*) in both approaches for single strip foundation but with slight changes in order. While for double-strip foundations in the factorial design approach, the most significant input parameter is *β* instead of *B* followed by *ϕ* and *b/B*.

## 6. Uncertainty measurements

In the literature, many approaches are available to check the uncertainty of predicted BC from the selected EPR models. In this work two approaches are explained:

1. Based on the Excel function = CONFIDENCE.NORM (Significant level, SD, Sample size), the uncertainty analysis results of the EPR models are presented in Table 11. The confidence interval indicates a range of possible values within which the real value may be found.
2. Using the following formula:
$$u=\sqrt{\frac{\sum (x_{i}-\bar{y}{)}^{2}}{n*(n-1)}}$$ (15)
  where, u = measurements uncertainty; x_i_ = i^th^ reading of data; ȳ = average of the data; and n = total numbers of the data.

**Table 11:** Uncertainty analysis of the EPR models.

Measuring index	Single foundation		Double foundations	
	Excel function	Eq 15	Excel function	Eq 15
Mean	2.7184	2.7184	1.9206	1.9206
Standard deviation	1.0111		0.8242	
Sample Size	1728	1728	1440	1440
Significant level	0.05		0.05	
Margin of error	0.0477	0.0243	0.0426	0.0217
Confidence Interval	2.7184 ± 0.0477	2.7184 ± 0.0243	1.9206 ± 0.0426	1.9206 ± 0.0217

The uncertainty of measurements is about 2.43%– 4.77% and 2.17%– 4.26% at a 95% confidence level for the selected models to predict the BC of single and double-strip foundations, respectively. These values indicate that the chosen models are capable of predicting and generating outcomes with reasonable and acceptable errors.

All input parameters of Eqs (13) and (14) must be within the data range used to construct the two EPR models mentioned to reduce the source of errors that may be obtained due to inaccurate simulation of the field conditions and improper boundary conditions in predicting the BC. Otherwise, the models must be validated to verify their accuracy and certainty.

## 7. Conclusions

This investigation presents two models using EPR techniques to predict the normalized bearing capacity *q*_*u*_/*γH* (corresponding to *5%B* settlement) from data obtained by FE simulation of the single and double strip foundations situated on the earth slope crest using different geometrical and geotechnical parameters such as (*B*, *c*, *ϕ*, *D*_*f*_*/B*, *β*, *b/B*, *S/B*). It is worth mentioning that the developed expressions are valid and accurate within the parameter value range considered in this study, but outside of these limits, the foretelling must be validated.

The following inferences are drawn from the analysis of the outcomes:

- In both cases (single and double foundations), the *q*_*u*_/*γ H* value increases as parameters *B*, *c*, *ϕ*, *D*_*f*_*/B*, *b/B*, *and S/B* increase, but it negatively correlates with *β*.
- For a single strip foundation, the bearing capacity improved noticeably up to *b/B* = 6, after which the improvement became insignificant, which means that the slope inclination influence vanished and the foundation behaved like it was placed on the horizontal ground.
- For double-strip foundations, the bearing capacity increases when the *S/B* ratio reaches 3–4; after that, the impact of interfering fades away and the failure pattern below the foundation is nearly like an individual one.
- The EPR technique offers the best capable models to forecast the BC of single and double-strip foundations situated on the slope crest, based on the different statistics criteria due to high score values achieved from the training, testing, and validation phases.
- Many evaluation criteria indicate that EPR models provide accurate prediction performance, and they can generate a simple mathematical equation that can be solved manually without the use of any software.
- Based on sensitivity analysis, the most important input parameters that impact the output results are *b/B*, *ϕ*, and *B* for single-strip foundations, and *β*, *ϕ*, *b/B*, and *B* for double-strip foundations placed on earthen slope crest.
- A mathematical expression has been developed based on the most appropriate EPR model to predict the BC of single and double-strip foundations placed on slope crests. This mathematical prophecy expression will serve as a simple and quick tool for geotechnical practicing and consulting engineers involved in hilly area planning and design.

## 8. Recommendations for future works

For further research works, the following can be addressed:

- Investigating the geotechnical effects of a similar layout and geometry of a strip foundation/s model on stability and safety at both the experimental and numerical scales.
- An extensive parametric study using the same footing models, but incorporating additional variables, such as slope height, soil anisotropy, amount of soil dilatancy, groundwater table, and varying constitutive models, to reduce potential errors and uncertainties associated with the model predictions is helpful.
- Developing new models using the EPR approach to incorporate other loading conditions, such as cyclic or earthquake-induced loading.

## Supporting information

S1 Dataset (XLSX)
